# Leveraging single-dose human papillomavirus vaccination dose-efficiency to attain cervical cancer elimination in resource-constrained settings

**DOI:** 10.1093/jncimonographs/lgae035

**Published:** 2024-11-12

**Authors:** Irene Man, Damien Georges, Partha Basu, Iacopo Baussano

**Affiliations:** Early Detection, Prevention and Infections Branch, International Agency for Research on Cancer, Lyon, France; Early Detection, Prevention and Infections Branch, International Agency for Research on Cancer, Lyon, France; Early Detection, Prevention and Infections Branch, International Agency for Research on Cancer, Lyon, France; Early Detection, Prevention and Infections Branch, International Agency for Research on Cancer, Lyon, France

## Abstract

**Background:**

In low- and middle-income countries, resource constraints remain a critical factor limiting access to cervical cancer preventive measures. The option of single-dose immunization could help improve access to human papillomavirus vaccination and attain cervical cancer elimination.

**Methods:**

With simulation models adapted to country-specific data and scenarios for single-dose protection derived from International Agency for Research on Cancer India vaccine trial data, we estimated the expected impact of single-dose vaccination in India, Rwanda, and Brazil, three countries with varying profiles of cervical cancer risk and vaccination timelines. In combination with single-dose vaccination, we explored different resource reallocation strategies based on dose efficiency, elimination attainment, and cervical cancer cases prevented, with the existing 2-dose program as a comparator.

**Results:**

Assuming lifelong single-dose protection, switching from 2-dose to 1-dose vaccination and reallocating resources to female catch-up could prevent 467-1336, 94-194, and 15-207 additional cervical cancer cases (per 100 000 women born) in cohorts aged 11-30 years in India, Rwanda, and Brazil, respectively. Resource reallocation to improve the current routine coverage could help eliminate cervical cancer in India and across all Brazilian states but not in Rwanda. For each country, we found a dose-efficient reallocation strategy (or a combination of strategies) together with 1-dose vaccination that could prevent more cervical cancers vs 2-dose vaccination, even in the worst-case scenario of single-dose protection.

**Conclusion:**

Adopting single-dose vaccination with resource reallocation is a resource-efficient approach to enhance progress toward cervical cancer elimination. The overall impact of vaccination can be maximized by fine-tuning resource reallocation to a country’s needs.

In resource-constrained settings, the burden of cervical cancer is disproportionately high and access to preventive measures limited (1). Although most high-income countries have already introduced human papillomavirus (HPV) vaccination, approximately 40% of low- and middle-income countries (LMICs) have not (as of 2023) (2), and approximately 73% of women living in LMICs, aged 30-49 years, have never undergone cervical cancer screening (as of 2022) (3). Unless the coverage of these preventive measures substantially improves, many LMICs will likely not reach the cervical cancer elimination threshold of 4 cases per 100 000 women-year, proposed by the World Health Organization (WHO) (4,5).

In this context, the 2022 update of the WHO recommendations to include the single-dose option of HPV could be a game changer (6). It has now been convincingly shown that a 1 dose of HPV vaccine elicits high-efficacy noninferior to 2 doses, for at least 3 years postvaccination, in the KENya Single-dose HPV-vaccine Efficacy (KEN SHE) Study vaccine trial, which includes randomized single-dose arms (of Cervarix and Gardasil-9) (7). These data corroborate studies with longer follow-up but no designed randomized single-dose arms, notably the Costa Rica vaccine trial’s 16-years data (on Cervarix) and the International Agency for Research on Cancer (IARC) India vaccine trial’s 15-years data (on Gardasil-4) being presented in this Monograph (8-11). Although longer single-dose protection durability data do not yet exist, immunological reasoning suggests that single-dose protection should be long lasting (12).

Since the 2022 recommendation update, 41 LMICs have adopted the single-dose strategy (2). Besides making introduction of vaccination easier, switching to single-dose vaccination could further enhance progress toward cervical cancer elimination if the resources saved on the second dose are reinvested in other scale-up interventions, such as expansion of vaccination target age or sex, coverage improvement in underserved populations, or improvement of cervical cancer screening.

Clearly, how best to reallocate the resources saved by adopting single-dose HPV vaccination may differ across settings. In this paper, taking India, Rwanda, and Brazil as 3 study cases with varying cervical cancer risk and vaccination introduction timelines, we explore how adopting single-dose HPV vaccination, combined with different resource reallocation strategies, could enhance the progress toward cervical cancer elimination, focusing on reallocation in catch-up of older female cohorts and coverage improvement in routine vaccination. Throughout, we account for uncertainty in single-dose long-term protection based on scenarios derived from IARC’s India vaccine trial data (8,13).

## Methods

### Simulation models

To simulate the impact of vaccination on HPV infection, we used a previously described HPV transmission model RHEA (14) and adapted it to India, Rwanda, and Brazil. RHEA is a population-based dynamic model describing the transmission of high-risk HPV types (16, 18, 31, 33, 35, 39, 45, 51, 52, 56, 58, 59, 68) through sex-, age- and risk group–dependent sexual contact. In brief, RHEA was parameterized by first deriving a part of the model’s sexual behavior parameters from survey data and subsequently calibrating the remaining sexual behavior parameters and HPV natural history parameters to fit to observed data age- and type-specific HPV prevalence in women in each country. With RHEA-estimated vaccination impact on HPV type–specific incidence, we then used a previously described cervical cancer progression model ATLAS (1) to estimate the impact on cervical cancer risk. ATLAS estimates the impact on the lifetime number of cervical cancer cases by cohort by discounting the baseline cervical cancer incidence with the estimated reduction in HPV incidence, weighted by HPV type–specific attributable fraction in cervical cancer while also accounting for death from other causes. See Supplementary Appendix A.1-3 (available online) for details of the models.

### Data sources

The models were constructed using the following country-specific data. Sexual behavior data for India and Rwanda were from the Demographic and Health Surveys (DHS) (15,16). DHS data for Brazil were relatively old. Hence, we used a more recent Brazilian survey (17). HPV prevalence was from the aforementioned Brazilian survey and other Indian and Rwandese surveys (18-20). Cervical cancer incidence was from Global Cancer Observatory (GLOBOCAN) (21). Vaccination coverage was from the WHO HPV Dashboard (2). For Brazil, we also used region-specific incidence from Brazilian National Cancer Institute (INCA) (22) and state-specific coverage data from Department of Informatics of the Unified Health System (DATASUS) (23) to simulate coverage improvement strategies restricted to some northern states with high burden (around 1.5 times national incidence) and low coverage (girls-boys coverage as low as 37%-15% in 1 state). Country- and age-specific mortality were from 2024 United Nations (UN) estimates. Country-specific attributable fractions were from a recent systematic review (24).

### Simulated strategies and scenarios

Using the constructed models, we simulated strategies for 1- or 2-dose vaccination in combination with possible resource reallocation, considering different scenarios for single-dose protection. Note that in general we use the term *strategies* for aspects policy makers can control and *scenarios* for aspects they cannot control.

As a reference, we considered the existing program in the 3 countries until now and assumed its continuation with 2-dose strategy in the future (see Table 1). In India, nationwide introduction of girls-only HPV vaccination with a locally produced quadrivalent vaccine is planned for 2025 (25). For simplicity, we disregarded past vaccination in the 2 states that had already introduced vaccination (only 2% of the population) (25). We assumed suboptimal coverage of 50% after introduction to mimic resource-constrained settings. As an alternative, we also considered 90% coverage (WHO target) (26). Rwanda introduced girls-only vaccination at age 12 years in 2011, with catch-up to approximately age 18 years (27). Brazil introduced girls-only vaccination for ages 9-14 years in 2014 and changed to gender-neutral vaccination in 2017 (2). Both Rwanda and Brazil use a quadrivalent vaccine and have consistent routine coverage of approximately 80% (2). For simplicity, we modeled routine vaccination to be given at exactly age 12 years in the 3 countries.

**Table 1.** Country profile of cervical cancer risk, prevention program, and resource savings under single-dose human papillomavirus (HPV) vaccination

Country profile	India	Rwanda	Brazil
Cervical cancer burden^a^			
Age-standardized incidence rate, per 100 000 women-year	17.7	18.9	12.7
Mortality, per 100 000 women-year	11.2	13.8	6.5
Cervical cancer screening^b^			
Organization	Opportunistic	Opportunistic	Opportunistic
Screening method	Visual inspection with acetic acid based	HPV- and visual inspection with acetic acid based	cytology-based
Screening coverage	2%	12%	42%
HPV vaccination^c^			
Year of national introduction in girls	2025 (anticipated)	2011	2013
Year of national introduction in boys	NA	NA	2017
National coverage in girls	Approximately 0% (only 2 states introduced vaccination)	82%	88%
National coverage in boys	NA	NA	62%
Vaccine type	Local quadrivalent vaccine (anticipated)	Gardasil-4	Gardasil-4
Dose schedule	2-dose (anticipated)	2-dose	2-dose
Resource saving with single dose^d^			
Vaccine doses	55 041 000	1 462 000	19 709 000
Vaccine and delivery costs, US$	$435 378 000 (assuming 50% girls-only coverage)	$11 564 000 (assuming above-mentioned coverage)	$155 898 000 (assuming above-mentioned coverage)

a Cervical cancer burden from Global Cancer Observatory (GLOBOCAN) (21). GLOBOCAN = Global Cancer Observatory; NA = not applicable; UN = United Nations.

b Organization and screening methods from CanScreen5 (43); coverage of screened in the last 5 years in women aged 35-49 years from Bruni et al. (3)

c Vaccination program from World Health Organization HPV Dashboard (2); Brazil coverage data from Department of Informatics of the Unified Health System (DATASUS) (23); most recent coverage data were used. DATASUS = Department of Informatics of the Unified Health System.

d Resource saved computed for upcoming 10 routine vaccinated cohorts assuming US$7.91 per-dose costs; United Nations (UN) data on cohort size (44).

Alternatively, we modeled switching to a single-dose strategy in 2025 combined with different resource reallocation strategies and compared the corresponding impact and dose efficiency. Firstly, we considered reallocation to a one-off single-dose catch-up campaign in females aged 11-30 years in 2025. Secondly, we considered reallocation to improve routine vaccination coverage. For Brazil, we also considered coverage improvement restricted to some northern states not expecting to reach elimination under the current coverage. We explored possible coverage between 0% and 100% for the described strategies.

Throughout, we assumed 2-dose quadrivalent vaccination to induce lifelong 95% efficacy against HPV 16 and 18 and 9% cross-protection for HPV 31, 33, and 45 based on existing trials’ data (scenario A) (8,28). Single-dose vaccination was simulated under 3 vaccine protection scenarios. We considered scenario A the most likely, that is, the same efficacy as 2-dose vaccination. For scenario B, we considered the same initial efficacies for HPV 16 and 18 as scenario A but waning to approximately 80% efficacy 20 years postvaccination. For scenario C, we considered lower initial efficacies at approximately 87.5% for HPV 16 and 18 [which is similar to the lower bound of the estimated efficacy in the KEN SHE trial (7)] and waning to approximately 75% efficacy 20 years postvaccination. For HPV 31, 33, and 45, we assumed the same waning rate of efficacy as HPV 16 and 18. Note that these efficacy scenarios were also assumed for women receiving catch-up vaccination at an older age. Lower effectiveness for vaccination at older age was captured in the model by assuming no vaccine effect on the clearance of already acquired infections. See Supplementary Appendix A.4 (available online) for the figures and derivation of the scenarios based on IARC’s India vaccine trial data (8,13).

Finally, we also explored the impact of a single-dose nonavalent vaccine, assuming high nonwaning efficacy of 95% for HPV 16, 18, 31, 33, 45, 52, and 58, in cases when a quadrivalent vaccine was not sufficient to attain cervical cancer elimination. This was done by estimating the nonavalent vaccine impact on the incidence of HPV 31, 33, 45, 52, and 58 with the quadrivalent vaccine impact on the incidence for HPV 16 and 18.

### Model outcomes

#### Resource savings

The number of resources saved by switching from 2- to 1-dose routine vaccination was estimated as the number of vaccine doses and the vaccination costs corresponding to the second dose in the 10 upcoming routine birth cohorts starting from 2025 (ie, cohorts born in years 2013-2023). We assumed that the resources saved could be spent any time within this 10-year period. We chose a 10-year period because it is sufficiently long to provide the required resources for the considered reallocation strategies and to generate definite single-dose durability data, although not too long for practical planning. Vaccination costs were computed with US$7.91 per-dose costs, which consist of US$4.50 Global Alliance for Vaccines and Immunisation (GAVI) vaccine price (29), US$0.50 supply costs (30), and US$2.91 delivery costs, an average of school-based program costs found in WHO delivery costs database (31).

#### Cervical cancer risk

We estimated lifetime cases of cervical cancer prevented in cohorts aged 0-30 years in 2025, which corresponded to the 10 routine cohorts for which resource savings were computed and the 20 cohorts for which catch-up was considered. To assess whether the routine coverage improvement strategies could enable elimination, we estimated the age-standardized incidence rate of cervical cancer [using the Segi world standard population (32)], evaluated in 100 years and compared it with the WHO elimination threshold (5). We did not assess the impact of catch-up on long-term age-standardized incidence rate, as catch-up mainly influences the time needed to reach long-term age-standardized incidence rate but not its level.

#### Dose efficiency

We estimated the dose efficiency of different resource reallocation strategies, defined as the number of vaccine doses needed to prevent 1 additional cervical cancer.

#### Composite strategy

Finally, to help decision makers prioritize and combine different reallocation strategies, we showcase for each country an example of composite strategies by successively adding the next most dose-efficient strategy in combination with switching to single-dose vaccination. For each country, we investigated whether there was a composite strategy using only the doses saved that could increase the total number of cervical cancer cases prevented compared with the 2-dose reference. In case such a composite strategy did not allow elimination, we also considered whether increasing the number of doses beyond those saved would do so.

## Results

### Resources saved with single-dose vaccination

Assuming 50% girls-only coverage in India, 82% girls-only in Rwanda, and 88% and 62% girls and boys coverage in Brazil, respectively, we found the following resource savings on the second dose in the upcoming 10 routine cohorts: 55, 1.5, and 20 million doses, and US$435, US$12, and US$156 million (Table 1). For India, savings increased to 99 million doses and US$783 million if we assume 90% girls-only coverage.

### Resource reallocation to older female catch-up

**Figure 1.**
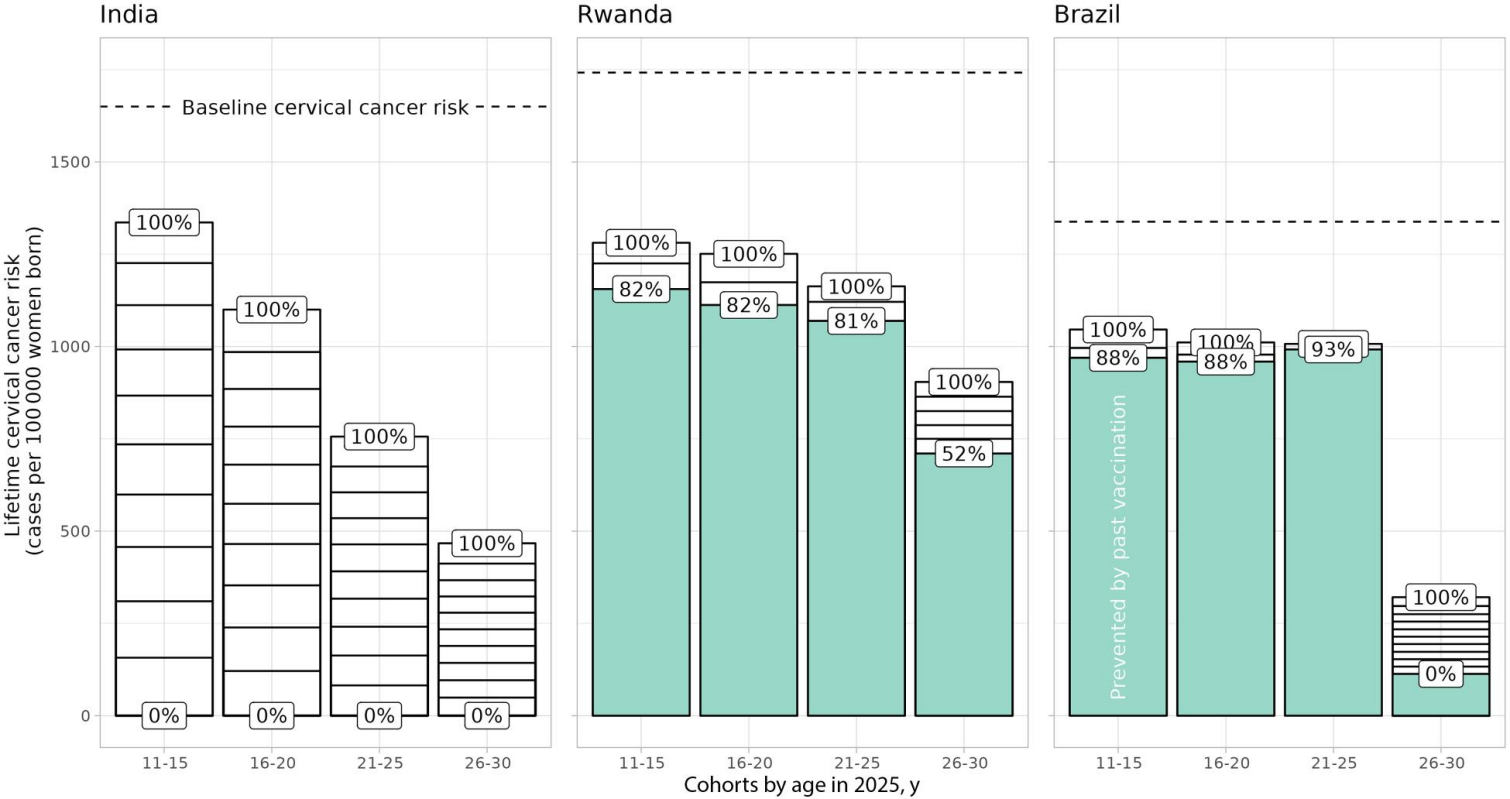
Impact of additional female catch-up in 2025 on lifetime cervical cancer risk by cohort. Results assuming scenario A of lifelong single-dose protection. **Total heights of bars:** maximum number of cervical cancer cases still preventable by catch-up assuming 100% coverage of a quadrivalent vaccine, with 1 block meaning 10% increase in catch-up coverage (larger block corresponds to better dose efficiency). **Green bars:** number of cervical cancer cases prevented by past vaccination. **Dashed lines:** baseline cervical cancer risk. **Percentage labels:** vaccination coverage. Note that, to ensure a uniform 5-year cohort layout, we also included the cohorts aged 11-12 years who are still awaiting their routine vaccination in 2025. For these cohorts, the impact from past vaccination (ie, **green bars**) was derived with the simulation in which these cohorts were vaccinated in the subsequent years, whereas the impact of with catch-up vaccination (ie, **white bars on top**) was derived with the simulation in which these cohorts were vaccinated already in 2025.

Figure 1 displays the impact of female catch-up in 2025 on lifetime cervical cancer risk by 5-year cohort (assuming lifelong single-dose protection scenario A), with 1) the total heights of the bars representing the maximum theoretical impact still preventable by catch-up assuming 100% coverage the quadrivalent vaccine, 2) little white blocks representing the impact of 10% increase of coverage, with larger blocks meaning more dose efficient, and 3) green bars representing the impact from past vaccination.

The maximum number of cervical cancer cases still preventable by catch-up vaccination decreased for increasingly older cohorts because of lower effectiveness in women who had already acquired infections. Overall, the maximum number of preventable cancer cases were the highest in India at 1336, 1100, 756, and 467 cancer cases (per 100 000 women born, in the successively older cohorts), then at 126, 139, 94, and 194 cancer cases in Rwanda, and then at 77, 52, 15, and 207 cancer cases in Brazil. The lower impact in Rwanda and Brazil was mainly because of the impact already achieved by past vaccination. Catch-up was also the most dose efficient in India at 69, 86, 126, and 217 number of vaccine doses needed to prevent 1 additional cervical cancer, then in Rwanda at 143, 130, 206, and 310 number of vaccine doses needed to prevent 1 additional cervical cancer, and then in Brazil at 156, 231, 461, and 426 number of vaccine doses needed to prevent 1 additional cervical cancer. The high dose efficiency in India was mainly because of the high attributable fraction of vaccine-targeted types and the low dose efficiency in Brazil because of the low baseline risk.

In the single-dose waning scenarios, the maximum impact and dose efficiency of catch-up deteriorated, but the overall pattern and ranking between countries remained (Supplementary Appendix Figure B1, available online).

### Resource reallocation to improve routine coverage

**Figure 2.**
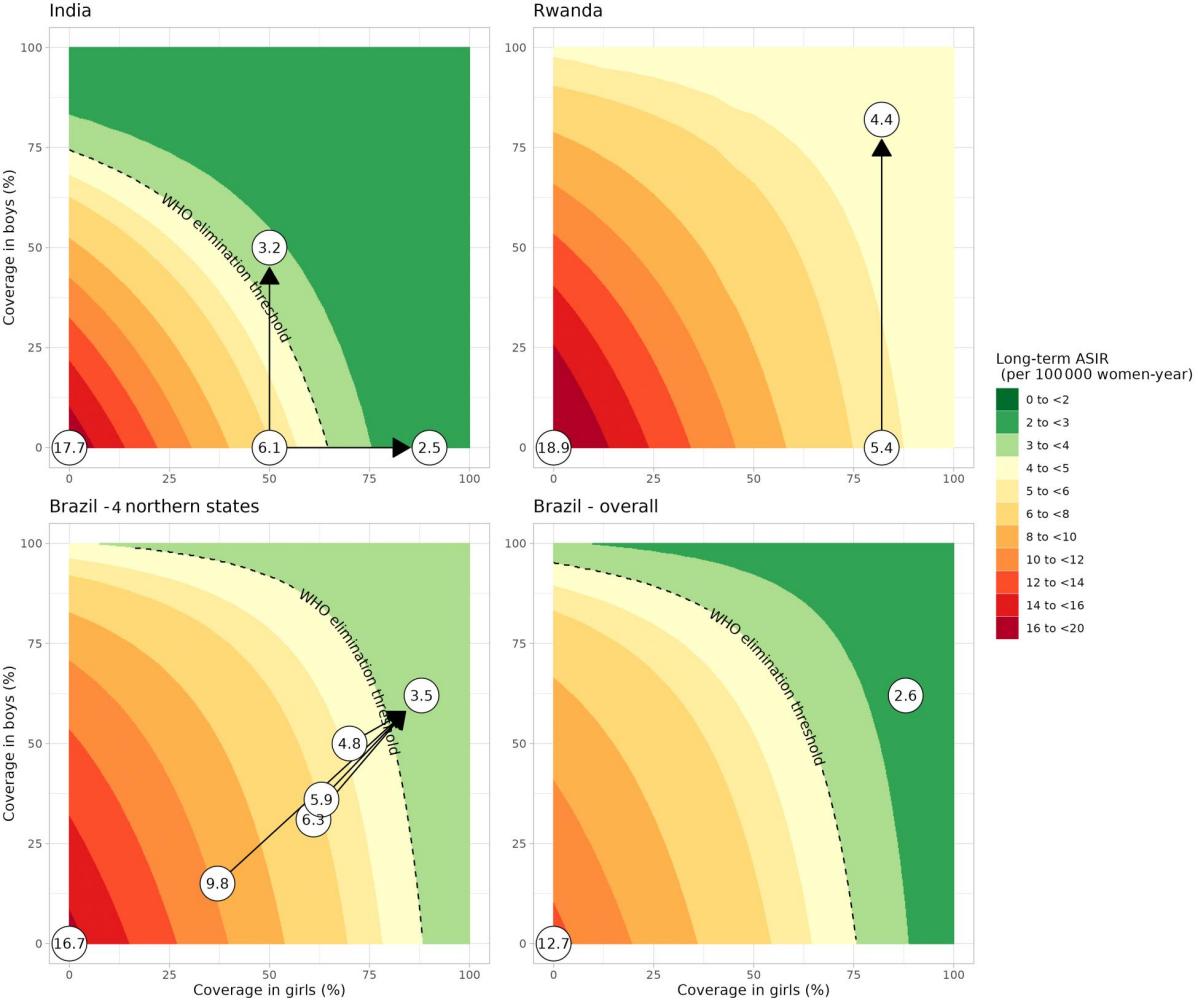
Expected long-term age-standardized incidence rate among women for combinations of coverage in girls and boys. Results assuming scenario A of lifelong single-dose protection and a quadrivalent vaccine. In panel Brazil – 4 northern states, we display the only 4 states throughout Brazil that are not expected to reach elimination under current coverage. **Arrows:** coverage improvement strategies. **Dashed lines:** World Health Organization cervical cancer elimination threshold. **Circles:** highlighted combinations of coverage with age-standardized incidence rate displayed within. ASIR = age-standardized incidence rate; WHO = World Health Organization.

Figure 2 displays the expected long-term age-standardized incidence rate under different sustained routine coverage in girls and boys (assuming lifelong single-dose protection scenario A), with 1) circles highlighting age-standardized incidence rate in selected scenarios (no vaccination, current coverage, and improved coverage), and 2) dashed lines representing the elimination threshold, which is reached with different combinations of vaccination coverage across countries as a result of different country profiles (eg, sexual behavior, HPV type–specific attributable fractions, and baseline risk).

For India, elimination was not attainable with 50% girls-only coverage (age-standardized incidence rate = 6.1 per 100 000 women-year). Coverage improvement in girls to 90% or boys to 50% enabled elimination (age-standardized incidence rate = 2.5 and 3.2, respectively). Elimination was harder to achieve for Rwanda. It was not attained with a quadrivalent vaccine under the current 82% girls-only coverage (age-standardized incidence rate = 5.4) neither was adding boys’ vaccination sufficient (age-standardized incidence rate = 4.4) or increasing girls’ coverage up to 100% sufficient. However, switching to a nonavalent vaccine could lead to elimination without increasing the current coverage (age-standardized incidence rate = 2; Supplementary Appendix Figure B2, available online). For Brazil, elimination was found to be attainable with the current vaccination coverage at the national level (age-standardized incidence rate = 2.6), except in 4 northern states with high burden and low coverage (age-standardized incidence rate = 4.8-9.8). However, coverage improvement in these states up to the national coverage levels could enable elimination (age-standardized incidence rate = 3.5).

For single-dose waning scenarios B and C, elimination attainment was unchanged under the reference coverage but did change for some scenarios (Supplementary Appendix Figure B3, available online). For India, switching to gender-neutral vaccination under 50% coverage was no longer sufficient for elimination, but improvement to 90% girls-only coverage still was. For Brazil, elimination attainment was unchanged at the national level, but in the 4 northern states, coverage above the national average was now needed to achieve elimination.

### Examples of dose-efficient composite reallocation strategies

**Figure 3.**
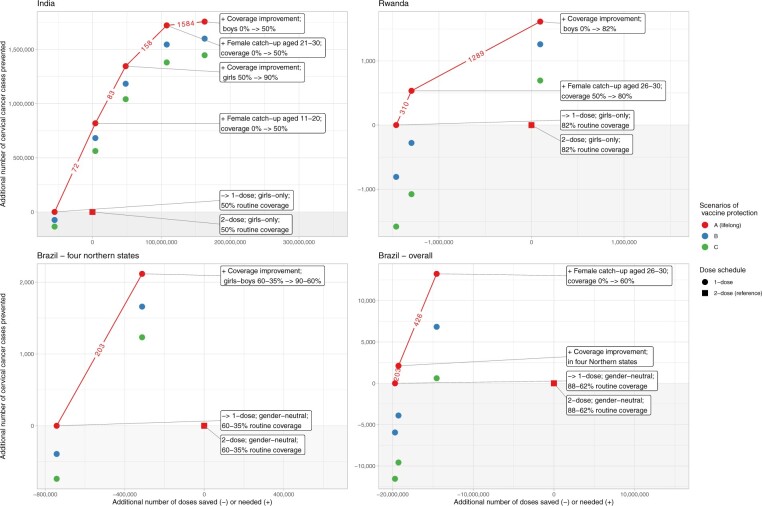
Composite resource allocation strategy based on ranked dose efficiency. In panel Brazil – 4 northern states, we display the only 4 states throughout Brazil that are not expected to reach elimination under current coverage. **Squares:** reference of 2-dose routine vaccination without any reallocation strategy. **Circles:** scenarios of 1-dose routine vaccination under successive addition of reallocation strategies. **Red-blue-green:** vaccine protection scenarios A-B-C. **Numbers on line segments:** dose efficiency, in number of additional vaccine doses needed to prevent 1 cervical cancer. **Grey area:** area of insufficient resource reinvestment leading to a decrease in overall number of cervical cancer cases prevented as compared with 2-dose reference. -> = to.

Figure 3 shows for each country an example of composite strategies constructed by successively adding the next most dose-efficient strategy in combination with switching to single-dose vaccination (depicted as collections of red lines) under coverage we deemed feasible. Resource requirements and health benefits of each separate strategy are described in Table 2.

**Table 2.** Overview of dose efficiency, resource requirement, and impact by reallocation strategy

Country		Strategy (ordered by dose efficiency)	Cohorts concerned	Coverage		Dose efficiency (in NND)^a^	Doses saved (−) or needed (+)	Gain (+) or loss (−) of cervical cancer cases prevented (by single-dose protection scenario)		
				Girls	Boys			A (no waning)	B	C
India	I	2-dose → single-dose	Upcoming 10 routine	50%	0%	NA^b^	−55 041 000	0	−74 171	−135 233
	II	Female catch-up	Women aged 11-20 years at 2025	0% → 50%	0%	72	+59 216 000	+ 818 988	+ 756 595	+ 698 000
	III	Coverage improvement in girls (to World Health Organization coverage target)	Upcoming 10 routine	50% → 90%	0%	83	+44 033 000	+ 527 542	+ 501 772	+ 478 900
	IV	Female catch-up	Women aged 21-30 years at 2025	0% → 50%	0%	158	+59 648 000	+ 376 609	+ 362 096	+ 339 138
	V	Coverage improvement in boys (girls-only → gender-neutral)	Upcoming 10 routine	50%	0% → 50%	1584	+55 041 000	+ 34 753	+ 54 429	+ 66 700
Rwanda	I	2-dose → single-dose	Upcoming 10 routine	82%	0%	NA^b^	−1 462 000	0	−808	−1583
	II	Female catch-up	Women aged 26-30 years at 2025	52% → 80%	0%	310	+165 000	+ 534	+ 530	+ 506
	III	Coverage improvement in boys (girls-only → gender-neutral)	Upcoming 10 routine	82%	0% → 82%	1289	+1 390 000	+ 1079	+ 1538	+ 1772
Brazil, 4 states	I	2-dose → single-dose	Upcoming 10 routine	60%	35%	NA^b^	−742 000	0	−393	−739
	II	Coverage improvement in both sexes (to national coverage)	Upcoming 10 routine	60% → 88%	35% → 62%	203	+429 000	+ 2117	+ 2053	+ 1972
Brazil, overall	I	2-dose → single-dose	Upcoming 10 routine	88%	62%	NA^b^	−19 709 000	0	−5946	−11 551
	II	Female catch-up	Women aged 26-30 years at 2025	0% → 60%	0%	426	+4 735 000	+ 11 127	+ 10 729	+ 10 189

a Dose efficiency was defined as “number of additional vaccine doses needed (NND) to prevent 1 cervical cancer case.” → = to; NA = not defined.

b Dose efficiency was not defined for strategy I “2-dose → single-dose” because no additional vaccine doses are needed.

For India, female catch-up for cohorts aged 11-20 years was most dose efficient (72 number of vaccine doses needed to prevent 1 additional cervical cancer). Such catch-up requires approximately the same number of doses saved if we assume the same coverage in the routine and catch-up cohorts, and it would already be able to increase the total number of cervical cancer cases prevented (Figure 3: the corresponding blue and green circles are above the grey area). If more resources are available, the next dose-efficient strategy to include would be coverage improvement in routine girls’ vaccination to 90% (83 number of vaccine doses needed) to achieve a level of coverage that should lead to cervical cancer elimination. After that, the next dose-efficient strategy would be female catch-up for cohorts aged 21-30 years (158 number of vaccine doses needed) then gender-neutral routine vaccination (1584 number of vaccine doses needed).

For Rwanda, female catch-up in cohorts up to age 25 years was most dose efficient, but we did not include this option, as we deemed it difficult to increase coverage above the current 80% coverage in these cohorts. The next dose-efficient strategy was female catch-up in cohorts aged 26-30 years (310 number of vaccine doses needed) where the current coverage is only 52%. In lifelong 1-dose scenario A, the number of cervical cancer cases prevented increased compared with the 2-dose reference, but it decreased in the waning scenarios because a large proportion of the second dose foregone was not reinvested. When also adding gender-neutral vaccination (1289 number of vaccine doses needed), the number of cervical cancer cases prevented increased above the 2-dose reference.

Lastly, for Brazil, coverage improvement in the states with the highest burden was most dose efficient (203 number of vaccine doses needed) and would allow elimination. This required approximately half of the doses saved in these states or 3% throughout the country. When only considering switching to single-dose vaccination in these states, the number of cervical cancer cases prevented always increased as compared with the 2-dose reference (Figure 3, Brazil – 4 northern states) but did not when considering Brazil overall (Figure 3, Brazil – overall). More resources would need to be reinvested to ensure an increase, for example, by adding female catch-up at ages 26-30 years with 60% coverage (426 number of vaccine doses needed). It should be noted that, still, a large part of the resources saved was left.

## Discussion

For countries where the burden of cervical cancer is still high and a large part of the population unvaccinated, the option of single-dose HPV vaccination holds great potential to strengthen the efforts toward cervical cancer elimination. In this modeling study, we investigated the expected impact of switching to single-dose vaccination in 3 prototypical countries—India, Rwanda, and Brazil—and searched for resource-efficient strategies to utilize the available resource and maximize overall vaccination impact.

Throughout the 3 countries, we found substantial resource savings by switching to single-dose routine HPV vaccination. The estimated amounts saved over 10 years were US$ 435 million, US$ 12 million, and US$ 156 million in India, Rwanda, and Brazil, respectively. In general, the higher the proportion of young unvaccinated women there are in a country, the easier it is to find an efficient and impact-expanding resource reallocation strategy. Countries where HPV vaccination has not or has only recently been introduced, such as India, are therefore typically countries that could benefit most from single-dose vaccination. Moreover, for the 3 countries, we found a suitable reallocation strategy, either through single or multiple interventions, to increase overall vaccination impact, even under the worst-case single-dose protection scenario.

The preferred reallocation strategy is, however, context specific and should be fine-tuned accordingly (as shown in Figure 3). In general, it is most impactful to first target still relatively young subpopulations in a country with low coverage and high burden, as shown for India. There are also subpopulations we did not consider that could benefit from catch-up, (eg, marginalized or hard-to-reach populations, migrants, and refugees) (33). Gender-neutral vaccination was found to be less dose efficient. However, it did help ensure an increase the overall vaccination impact, hence, acting as a resilience strategy against possible reduced single-dose protection, as shown for Rwanda.

There are resource reallocation strategies we did not consider that could also increase the overall impact of cervical cancer prevention. For example Rwanda, all considered strategies with a quadrivalent vaccine were insufficient for cervical cancer elimination because of the relatively low attributable fraction of the vaccine-targeted types: 73.7% in Rwanda vs 79.6% in Brazil and 84.9% in India (24). If an affordable vaccine price can be negotiated, reallocating resources to introduce a higher-valent vaccine could be an interesting option, which we showed could lead to cervical cancer elimination (Supplementary Appendix Figure B2, available online). As another example, in Bhutan (not modeled here), where HPV vaccination has been introduced for a long time, with catch-up, high coverage, and even gender-neutral vaccination (2), little margin is left to expand on vaccination. In this context, resources could better be reallocated to support ongoing and efficient HPV-based screening (34).

Some modeling assumptions we made are worth discussing. For Brazil, we used a national coverage of 88% and 62% in girls and boys, which is different to the coverage on WHO HPV dashboard. The latter coverage is lower because it is based on a cross-sectional average among all girls aged 9-14 years (2). Such an average is suitable for monitoring trends of coverage; however, for the purpose of modeling the impact of the vaccination, it was better to use the cumulative coverage at age 14 years. Also, for HPV 31, 33, and 45, we assumed a 9% initial efficacy and diminution over time based on IARC’s India vaccine trial data on Gardisil-4 (13). These assumptions could be compatible with other newly emerged vaccines targeting HPV 16 and 18 (35) for which also little evidence exists on cross-protection with a single-dose schedule. To meet the global vaccine demand required for cervical cancer elimination, it will be crucial to consider these other vaccines administered with a single dose.

The main limitation of this study was the use of dose efficiency to assess resource utilization. This measure allows for straightforward interpretation but does not account for certain aspects that differ between strategies. For instance, catch-up of older cohorts or populations living in remote areas is often costlier than school-based routine vaccination (31,36). However, because of the lack of comprehensive delivery cost data by age and type of outreach delivery method, we opted for a dose efficiency–based approach, which was also used in various previous studies modeling the impact of single-dose vaccination (37,38). Moreover, this approach was sufficient to show that in most countries, switching to single-dose vaccination in combination with resource reallocation could increase the overall impact of vaccination. If, for a given country, more detailed advice is needed and it is not obvious which intervention is most appropriate, it would be helpful to conduct a formal cost-effectiveness analysis provided local costs data are available.

Our results complement and expand previous modeling studies assessing the impact of single-dose vaccination. We confirm the conclusion drawn by previous studies that given immunization with 1 dose of HPV vaccine, revaccinating the same cohorts with a second dose would be less efficient (37-40), unless there is an excessively short duration of single-dose vaccine protection, which is unlikely given the latest evidence (7-11). Among the previous single-dose modeling studies, Drolet et al. (37) also suggests that reallocating the saved dose to target unvaccinated populations could be more efficient. Here, by explicitly modeling resource constraints across different strategies, we show that catch-up in older women, possibly up to age 30 years, could be an efficient strategy. Furthermore, we showcased the need to consider reallocating resources to a nonavalent vaccine in settings with high cervical cancer incidence.

Finally, there are context-specific aspects besides efficiency that need to be considered to ensure adapted health policies, including local equity, cultural acceptability, and ease of implementation. Some resource-demanding interventions can be found worthwhile when viewed from a wider and longer-term perspective. A good example is gender-neutral vaccination; it is often in line with many societal values, easy to implement, and helps increase the resilience of cancer prevention against fluctuating coverage (41). Furthermore, some resource constraints can be mitigated through political commitment and creative solutions (eg, cofinancing, donation, price negotiation). Eventually, the fastest way toward global cervical cancer elimination will rely on innovative ways to utilize available resources while aiming for maximal impact (42).

## Supplementary Material

lgae035_Supplementary_Data

## Data Availability

The following data were from publicly available sources: sexual behavior data for India and Rwanda from DHS; cervical cancer incidence from GLOBOCAN; Brazilian region-specific cervical cancer incidence from INCA, vaccination coverage data from WHO HPV Dashboard; Brazilian vaccination coverage data from DATASUS; cervical cancer attributable fraction from a recent system review. The following data were from personal communication: HPV vaccination delivery costs shared by Paul Bloem and Karene Yeung from WHO Department of Immunization Vaccine and Biological; POP-Brazil sexual behavior and HPV prevalence data shared by Eliana Wendland from the Federal University of Health Sciences of Porto Alegre.
