# Supplementary material for: Leveraging single-dose human papillomavirus vaccination dose-efficiency to attain cervical cancer elimination in resource-constrained settings
Source: J Natl Cancer Inst Monogr. 2024 Nov 12;2024(67):400–9. doi: 10.1093/jncimonographs/lgae035 (PMC11555267; doi:10.1093/jncimonographs/lgae035)
Supplement: lgae035_Supplementary_Data [file lgae035_supplementary_data.docx]

**Appendices**

Contents

[List of tables 1](#_Toc162603378)

[List of figures 1](#_Toc162603379)

[A.1. HPV Transmission model 2](#_Toc162603380)

[A.1.1. Demography 2](#_Toc162603381)

[A.1.2. Sexual contact behaviour 2](#_Toc162603382)

[A.1.3. HPV natural history 3](#_Toc162603383)

[A.1.4. HPV vaccination 4](#_Toc162603384)

[A.1.5. Model equations 5](#_Toc162603385)

[A.2. Model calibration 8](#_Toc162603386)

[A.2.1. Calibration to sexual behaviour data 8](#_Toc162603387)

[A.2.2. Calibration to HPV prevalence data 10](#_Toc162603388)

[A.3. Computation of model outcomes 14](#_Toc162603389)

[A.3.1. Impact on HPV cumulative incidence 14](#_Toc162603390)

[A.3.2. Impact on number of cervical cancer cases 14](#_Toc162603391)

[A.4. Single-dose vaccine protection scenarios 16](#_Toc162603392)

[A.5. HPV-FRAME checklist 17](#_Toc162603393)

[B.1. Supplementary results in figures 19](#_Toc162603394)

[Reference 23](#_Toc162603395)

# List of tables

Table A1. List of model parameters

Table A2. Female all-cause mortality rates

Table A3. Cervical cancer incidence

Table A4. HPV-type-specific attributable fractions to cervical cancer

Table A5. Overview of parameters under different vaccine protection assumptions

Table A6. HPV-FRAME checklist

# List of figures

Figure A1. Compartments in RHEA modelling the HPV natural history in unvaccinated and vaccinated people

Figure A2. HPV natural history in RHEA regarding the CIN stages modelled within the Infected compartment

Figure A3. Mixing patterns in women at selected ages derived from sexual behaviour survey data

Figure A4. Partner acquisition rates derived from sexual behaviour survey data

Figure A5. Model fit of the type-specific HPV prevalence data

Figure A6. Calibrated assortativeness parameters and transmission probabilities

Figure A7. Scenarios of single-dose vaccine efficacy by HPV type

Figure B1. Impact of additional female catch-up in 2025 on lifetime cervical cancer risk in waning scenarios

Figure B2. Expected long-term ASIR among for combinations of coverage in girls and boys with a nonavalent vaccine

Figure B3. Expected long-term ASIR among for combinations of coverage in girls and boys in waning scenarios

**Appendix A**

# A.1. HPV Transmission model

For this study, we adapted a previously published HPV transmission model,[1] also called RHEA. The model is dynamic and population-based, which is also referred to as compartmental. This model is part of the modelling platform METHIS (ModElling plaTform for Hpv Infection-related cancers: an open-Source tool), developed by the Public Health Decision Modelling Team at The International Agency for Research on Cancer.[2] The model is implemented in C, which allows fast simulation, and is equipped with an interface in R.

## A.1.1. Demography

The model describes an open population with an age of entrance of 10 years and a maximum age of 80 years. The population is divided into strata of age and sex, denoted by $a\in[10, 80)$ and $g\in\{W,M\}$, respectively. At any given time $t$, the proportion of the population of sex $g$ at age $a$ is denoted by $N^{g}(a,t)$.

Death occurs according to sex- and age-specific rate $m^{g}(a)$. Influx rate into the population per time step due to birth is given by $b$, which is set to a value that keeps the total population constant over time. A constant age-specific population size distribution is achieved through the simulation of a burn-in period. New-borns are divided the strata of male and female population with a 50%-50% distribution.

See **Table A1** for the values and references of the used demographic parameters.

## A.1.2. Sexual contact behaviour

People entering the population are further stratified into three Classes Sexual Activity, CSAs in short, and indexed by $l\in\{low,medium,high\}$, according to sex-specific distribution $p^{g}(l)$. For this work, we fixed the distribution to $\{p^{g}\left( low \right),p^{g}\left( medium \right),p^{g}\left( high \right)\}=\{0.80, 0.15, 0.05\}$. The assigned CSA is kept throughout lifetime. The proportion of the population belonging to sex $g$, age $a$, and CSA $l$ at time $t$ is denoted by $N^{g}\left( a,l, t \right)$.

Together with sex and age, CSA determines rates of partner acquisition $c^{g}(a, l)$. In the model, we only implement partnerships between opposite sex and hence heterosexual contact only. In addition, assortativeness of partner mixing is governed by a sex-specific mixing matrix for age $\rho^{g,age}(a,a')$and a sex-specific mixing matrix for CSAs $\rho^{g,csa}(l, l')$. For $\rho^{g,age}(a,a')$, the row of a given age $a$ gives the distribution of how people aged$a$ select the age of their partner and hence sums up to unity. The same holds for each row of $\rho^{g,csa}(l, l')$ to give the distribution across CSAs. The values of the described sexual behaviour parameter, $c^{g}\left( a, l \right)$, $\rho^{g,age}\left( a, a^{'} \right)$, and $\rho^{g,csa}(l, l')$, were derived from sexual behaviour survey data through parametrization steps described in **Section A.2.1**.

Partner acquisition rates and mixing matrices derived from sexual behaviour survey data could be subjected to bias. To adjust for possible bias in assortative patterns, there are two pairs of sex-specific assortativeness adjustment parameters, $\varepsilon^{g, age}$ and $\varepsilon^{g,csa}$. These parameters provide the weights for the from the data derived mixing matrices, on one hand, and proportionate mixing matrices, on the other, in creating the final mixing matrix $\tilde{\rho}^{g}\left( a, a^{'},l, l^{'},t \right)$, which captures the mixing between any age strata, from $a$ to $a'$, and between any CSA strata, from $l$ to $l'$, at a given time $t$ for sex $g$. The exact formula is given by

$$\tilde{\rho}^{g}\left( a, a^{'},l, l^{'},t \right)=A\cdot B,$$

where

$$A=\varepsilon^{g, age}\frac{\sum_{csa} N^{g^{'}}(a',csa,t)\cdot c^{g^{'}}(a',csa)}{\sum_{age} \sum_{csa} N^{g^{'}}(age,csa,t)\cdot c^{g^{'}}(age,csa)}+\left( 1-\varepsilon^{g, age} \right)\rho^{g,age}\left( a,a^{'} \right),$$

$$B=\varepsilon^{g, csa}\frac{N^{g^{'}}(a',l',t)\cdot c^{g^{'}}(a',l')}{\sum_{csa} N^{g^{'}}(a',csa,t)\cdot c^{g^{'}}(a',csa)}+\left( 1-\varepsilon^{g, csa} \right)\rho^{g,csa}\left( l,l^{'} \right).$$

The value of the age assortativeness adjustment parameters $\varepsilon^{g, age}$ should be between zero and one. When being one, mixing is completely proportionate, and when being zero, mixing occurs according to the data-derived mixing matrices. As for the CSA assortativeness adjustment parameters $\varepsilon^{g, csa}$, being one is completely proportionate, and being zero is mixing only within each CSA.

Furthermore, to ensure the supply and demand in partners prescribed by the data-derived partner acquisition rates $c^{g}(a,l)$ are balanced between each pair of strata of age, sex, and CSA, the discrepancy in partner acquisition rates $\Delta(a, a^{'}, l, l',t)$ are computed and adjusted for as follows:

$$\Delta\left( a, a^{'}, l, l^{'},t \right)=\frac{N^{W}(a,l,t)\cdot c^{W}(a,l)\cdot\tilde{\rho}^{W}(a, a^{'},l, l',t)}{N^{M}(a',l',t)\cdot c^{M}(a',l')\cdot\tilde{\rho}^{M}(a', a,l', l,t)},$$

$\tilde{c}^{W}\left( a, a^{'}, l, l^{'},t \right)=c^{W}\left( a, l \right)\cdot{\Delta\left( a, a^{'}, l, l^{'},t \right)}^{-(1-\theta)},$

$$\tilde{c}^{M}\left( a, a^{'}, l, l^{'},t \right)=c^{M}\left( a,l \right)\cdot{\Delta\left( a^{'}, a, l', l,t \right)}^{\theta}.$$

With these adjusted partner acquisition rates, balanced supply and demand of contact between each pair of strata hold:

$N^{W}(a,l,t)\cdot\tilde{c}^{W}\left( a, a^{'}, l, l^{'},t \right)\cdot\tilde{\rho}^{W}(a, a^{'},l, l',t)=N^{M}(a',l',t)\cdot\tilde{c}^{M}\left( a^{'},a, l^{'},l,t \right)\cdot\tilde{\rho}^{M}(a^{'}a,l^{'},l,t)$.

For this study, $\theta$ was set to 0.5 so that parameters for men and women are altered at the same degree.

## A.1.3. HPV natural history

Thirteen high-risk (HR) HPV types are included in the model: HPV16, 18, 31, 33, 45, 35, 39, 51, 52, 56, 58, 59, and 68. The modelled HPV types are assumed to be transmitted independently, governed by type-specific natural history parameters. Natural history parameters include parameters related to the probability of transmission, duration of infection and natural immunity. People enter the population uninfected and are susceptible to HPV infection. In the absence of vaccination, the natural history of all HR HPV types is assumed to follow the “Susceptible-Infected-Immune/Removed-Susceptible” dynamics in women. See the column “Unvaccinated” in **Figure A1**. In men, it has generally been observed that the HPV prevalence and the rate of acquiring new HPV infection remain constant across age, and that the seroconversion rate is low after natural infection.[3-5] Hence, we assumed the “Susceptible-Infected-Susceptible” dynamics in men. The dynamics in men also can be imagined as the one in women with an infinitely short Immune/Removed state.

**Figure A1. Compartments in RHEA modelling the HPV natural history in unvaccinated and vaccinated people.** $c$: vaccination coverage; ${VE}_{i}$: vaccine efficacy of type $i$; $\varphi_{i}$: rate of waning vaccine protection.


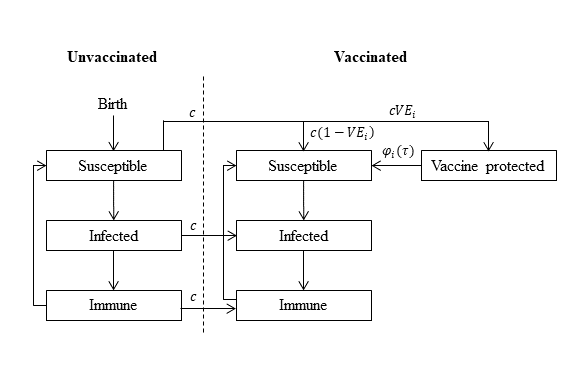


For unvaccinated susceptibles, infections with a given HPV type $i$ are acquired according a dynamic rate, which is also called the force of infection. Besides changing over time, this rate $\lambda_{i}^{g}(a,l,t)$ also depends on age, gender, age, and CSA because contact rates and choice of partner depend on these variables. The force of infection also depends on the type-specific transmission probability $\beta_{i}\in(0,1)$ and increases with the number of infected people in the population at any given time point. The exact expression for the force of infection is given by

$$\lambda_{i}^{g}\left( a,l,t \right)=\beta_{i}\cdot\sum_{a'} \sum_{l'} \left[ \tilde{c}^{g}\left( a, a^{'}, l, l',t \right)\cdot\tilde{\rho}^{g}\left( a, a^{'},l, l^{'},t \right)\cdot\left( \frac{I^{g^{'}}(a^{'},l^{'},t)}{N^{g'}(a',l',t)} \right) \right],$$

where $I^{g^{'}}(a^{'},l^{'},t)$ is the proportion of infected people at time $t$ of the opposite sex $g^{'}$, at age $a^{'}$, and in CSA $l^{'}$.

After acquisition, the duration of infection follows the clearance distribution $\chi_{i}(\tau)$, which depends on the duration of time since acquisition $\tau,$and is described by six type-specific parameters $\gamma_{i}, \eta_{i}, \delta_{i,1}, \delta_{i,2}, \nu_{i,1}, \nu_{i,2}$. This distribution describes the process of infection through stages of CIN0, CIN1, regressive CIN2/3, and non-regressive CIN2/3 stages, as modelled in an extensively validated cervical cancer progression model.[6, 7] See **Figure A2** for the stages of infection. Non-regressive CIN2/3 here represents the part of HPV infections that will persist and progress to cancer, whereas regressive CIN2/3 does clear eventually. For those HPV infections that do not persist, upon clearance, men become susceptible and women become Removed/Immune. Note that while the clearance distribution of HPV infection in the model was constructed by reproducing the exact transitions through the different CIN stages, we do not explicitly simulating the prevalence or incidence of these intermediate stages to limit the computational load of simulation.

The duration of natural immunity in women is exponentially distributed with type-specific rate $\mu_{i}$. After natural immunity has waned, women become susceptible again. For men, $\mu_{i}$ is essentially infinite as we assume no natural immunity. All natural history parameters are based on a previously validated model [6, 7], except the probabilities of transmission, which are obtained through the calibration procedure described in **Section A.2.2**.

**Figure A2. HPV natural history in RHEA regarding the CIN stages modelled within the Infected compartment.**

$$\delta_{i,1}$$

$$\nu_{i,1}$$

$$\eta_{i}$$

$$\lambda_{i}^{g}(a,l,t)$$

$$\delta_{i,2}$$

$$\gamma_{i}$$

Non-regressive
CIN2/3

Regressive
CIN2/3

$$\nu_{i,2}$$

CIN1

CIN0

Infected

Effects of HIV, smoking, and use of contraceptives, which are also risk factors of HPV transmission and cervical cancer progression, are not included in the model. For HIV infection, this choice was based on the relatively low HIV prevalence in India (0.2%), Rwanda (3.32%), and Brazil (0.37%) (in females aged 15 years or older.[8] As for smoking and the use of contraceptives, the choice was based on the lack of data.

## A.1.4. HPV vaccination

HPV vaccination can be applied to the unvaccinated part of the population at any given time $t$ for any given sex $g$, age $a$, and CSA $l$ with specific vaccination coverage $c^{g}\left( a,l,t \right)$. Given a type-specific efficacy of ${VE}_{i}$, an all-or-nothing or a degree working mechanism can be used to model vaccine protection. For this work, we chose the implementation with an all-or-nothing working mechanism. This means that, when an amount of $c^{g}\left( a,l,t \right)$ people are vaccinated, an amount of $c^{g}\left( a,l,t \right)\cdot{VE}_{i}$ people are vaccinated and transit to the compartment of vaccinated and vaccine-protected (the most right column in **Figure A1**), whereas an amount of $c^{g}\left( a,l,t \right)\cdot(1-{VE}_{i})$ people transit to the compartment of vaccinated but not vaccine-protected (the middle column in **Figure A1**). For simplicity, we assumed that none of the already infected or already immune (by natural infections) people receive vaccine protection. Vaccinated people who are not vaccine-protected continue to follow the same HPV natural history as described for the unvaccinated people. Finally, we model waning of vaccine protection with a rate $\varphi_{i}(\tau)$ that depends on the time since vaccination $\tau$. After vaccine protection has waned, these people transit to the compartment of the people vaccinated but not vaccine protected and susceptible (the middle column in **Figure A1**).

## A.1.5. Model equations

Before giving the system of equations governing the transitions between compartments in the model, we introduce some notations. The notations of compartments for a given sex $g$, age$a$, CSA $l$ at time $t$ are as follows.

- Unvaccinated, susceptible $S^{u,g}\left( a,l,t \right)$
- Unvaccinated, infected for time $\tau$ $I^{u,g}\left( a,l,t,\tau\right)$
- Unvaccinated, immune (by natural infection) $R^{u,g}\left( a,l,t \right)$
- Vaccinated but not vaccine-protected, susceptible $S^{v,g}\left( a,l,t \right)$
- Vaccinated but not vaccine-protected, infected for time $\tau$ $I^{v,g}\left( a,l,t,\tau\right)$
- Vaccinated but not vaccine-protected, immune (by natural infection) $R^{v,g}\left( a,l,t \right)$
- Vaccinated for time $\tau$ and vaccine-protected $V^{g}\left( a,l,t,\tau\right)$
- Infected regardless of for how long and vaccination status $I^{g}\left( a,l,t \right)=\sum_{\tau} \left[ I^{u,g}\left( a,l,t,\tau\right)+I^{v,g}(a,l,t,\tau) \right]$

At any given time $t$, the population size of a given sex $g$, age$a$, CSA $l$ is as follows.

- $N^{g}\left( a,l,t \right)=S^{u,g}\left( a,l,t \right)+\sum_{\tau} I^{u,g}\left( a,l,t,\tau\right)+R^{u,g}\left( a,l,t \right)+S^{v,g}\left( a,l,t \right)+\sum_{\tau} I^{v,g}\left( a,l,t,\tau\right)+R^{v,g}\left( a,l,t \right)+\sum_{\tau} V^{g}\left( a,l,t,\tau\right)$

The model is governed by the following boundary conditions.

- $S^{u,g}\left( 10,l,t \right)=b\cdot p^{g}(l)$
- $I^{u,g}\left( 10,l,t,\tau\right)=I^{v,g}\left( 10,l,t,\tau\right)=R^{u,g}\left( 10,l,t \right)=R^{v,g}\left( 10,l,t \right)=0$
- $I^{u,g}\left( a,l,t,0 \right)=\lambda_{i}^{g}\left( a,l,t \right)\cdot S^{u,g}\left( a,l,t \right)$
- $I^{v,g}\left( a,l,t,0 \right)=\lambda_{i}^{g}\left( a,l,t \right)\cdot S^{v,g}\left( a,l,t \right)$
- $V^{g}\left( a,l,t,0 \right)=c^{g}\left( a,l,t \right)\cdot{VE}_{i}\cdot S^{u,g}\left( a,l,t \right)$

These boundary conditions define together with the following set of equations the model.

- $\frac{\partial S^{u,g}\left( a,l,t \right)}{\partial a}+\frac{\partial S^{u,g}\left( a,l,t \right)}{\partial t}=\mu_{i}\cdot R^{u,g}\left( a,l,t \right)-(\lambda_{i}^{g}\left( a,l,t \right)+c^{g}\left( a,l,t \right)+m^{g}\left( a \right))\cdot S^{u,g}\left( a,l,t \right)$
- $\frac{\partial I^{u,g}\left( a,l,t,\tau\right)}{\partial a}+\frac{\partial I^{u,g}\left( a,l,t,\tau\right)}{\partial t}+\frac{\partial I^{u,g}\left( a,l,t,\tau\right)}{\partial\tau}=-(\chi\left( \tau\right)+c^{g}\left( a,l,t \right)+m^{g}\left( a \right))\cdot I^{u,g}\left( a,l,t,\tau\right)$
- $\frac{\partial R^{u,g}\left( a,l,t \right)}{\partial a}+\frac{\partial R^{u,g}\left( a,l,t \right)}{\partial t}=\sum_{\tau} \chi\left( \tau\right)\cdot I^{u,g}(a,l,t,\tau)-(\mu_{i}+c^{g}\left( a,l,t \right)+m^{g}\left( a \right))\cdot R^{u,g}\left( a,l,t \right)$
- $\frac{\partial S^{v,g}\left( a,l,t \right)}{\partial a}+\frac{\partial S^{v,g}\left( a,l,t \right)}{\partial t}=\mu_{i}\cdot R^{v,g}\left( a,l,t \right)+c^{g}\left( a,l,t \right)\cdot\left( 1-{VE}_{i} \right)\cdot S^{u,g}\left( a,l,t \right)+\sum_{\tau} \varphi_{i}(\tau)\cdot V^{g}(a,l,t,\tau)-(\lambda_{i}^{g}\left( a,l,t \right)+m^{g}\left( a \right))\cdot S^{u,g}\left( a,l,t \right)$
- $\frac{\partial I^{v,g}\left( a,l,t,\tau\right)}{\partial a}+\frac{\partial I^{v,g}\left( a,l,t,\tau\right)}{\partial t}+\frac{\partial I^{v,g}\left( a,l,t,\tau\right)}{\partial\tau}=c^{g}\left( a,l,t \right)\cdot I^{u,g}\left( a,l,t,\tau\right)-(\chi\left( \tau\right)+m^{g}\left( a \right))\cdot I^{v,g}\left( a,l,t,\tau\right)$
- $\frac{\partial R^{v,g}\left( a,l,t \right)}{\partial a}+\frac{\partial R^{v,g}\left( a,l,t \right)}{\partial t}=\sum_{\tau} \chi\left( \tau\right)\cdot I^{v,g}(a,l,t,\tau)+c^{g}\left( a,l,t \right)\cdot R^{u,g}\left( a,l,t \right)-(\mu_{i}+m^{g}\left( a \right))\cdot R^{v,g}\left( a,l,t \right)$
- $\frac{\partial V^{g}\left( a,l,t,\tau\right)}{\partial a}+\frac{\partial V^{g}\left( a,l,t,\tau\right)}{\partial t}+\frac{\partial V^{g}\left( a,l,t,\tau\right)}{\partial\tau}=-(\varphi_{i}(\tau)+m^{g}\left( a \right))\cdot V^{g}\left( a,l,t,\tau\right)$

**Table A1. List of model parameters.**

| **Notation** | **Description** | **Values / ranges** | **Reference** |
| --- | --- | --- | --- |
| *Demography* | | | |
| $m^{W}(a)$ ,$m^{M}(a)$ | Gender- and age-specific mortality | **Table A2** | UN [9] |
| $b$ | Population sex-specific birth rate | Set such that the total population stays constant given $m^{g}(a)$ and 50%-50% distribution of female and male population influx | NA |
| *Sexual contact behaviour* | | | |
| $p^{W}\left( low \right),p^{W}\left( medium \right),p^{W}\left( high \right),$  $p^{M}\left( low \right),p^{M}\left( medium \right),p^{M}\left( high \right)$ | Population distribution of CSA | 0.80, 0.15, 0.05,  0.80, 0.15, 0.05 by assumption | NA |
| $\rho^{W,age}(a,a')$ | Age mixing matrix in women | **Figure A3** | Survey data on the age difference between sexual partners.[10-12] |
| $\rho^{M,age}(a,a')$ | Age mixing matrix in men | Derived from the age matrix in women by transposing it and then rescaling rows to unity. | NA |
| $c^{W}(a)$, $c^{M}(a)$ | Partner acquisition rate in women and men | **Figure A4** | Survey data on the number of sexual partners by age.[10-12] |
| $\rho^{W,csa}(l, l')$, $\rho^{M,csa}(l, l')$ | CSA mixing matrix in women and men | Identity matrix by assumption | NA |
| $\varepsilon^{W, age}$, $\varepsilon^{M,age}$ | Age assortativeness adjustment parameters | Derived from model calibration with the following range for candidate values: Uniform(0.1, 0.9). | NA |
| $\varepsilon^{W, csa}$, $\varepsilon^{M,csa}$ | CSA assortativeness adjustment parameters | Derived from model calibration with the following range for candidate values: Uniform(0.1, 0.9). | NA |
| $\theta$ | Balance parameter | 0.5 by assumption | NA |
| *HPV natural history* | | | |
| $\beta_{16}, \beta_{18},\beta_{nona}, \beta_{other}$ | Transmission probability of type $i$ per sex act | Derived from model calibration with the following range for candidate values: Uniform(0.1, 0.9). | NA |
| $\gamma_{16}, \gamma_{18},\gamma_{nona}, \gamma_{other}$ | Clearance rate from CIN0 | 0.824 per year for HPV 16,  0.955 per year for HPV 18,  1.18 per year for HPV 31/33/45/52/58 and remaining HR HPV types | [6, 7] |
| $\eta_{16}, \eta_{18},\eta_{nona}, \eta_{other}$ | Progression rate from CIN0 to CIN1 | 0.676 per year for HPV 16,  0.545 per year for HPV 18,  0.324 per year for HPV 31/33/45/52/58 and remaining HR HPV types | [6, 7] |
| $\delta_{16,1}, \delta_{18,1}$,$\delta_{nona,1}$, $\delta_{other,1}$ | Clearance rate from CIN1 | 0.133 per year for HPV 16,  0.386 per year for HPV 18,  0.481 per year for HPV 31/33/45/52/58 and remaining HR HPV types | [6, 7] |
| $\delta_{16,2}$,$\delta_{18,2},\delta_{nona,2}$,$\delta_{other,2}$ | Clearance rate from regressive CIN2/3 | 2.10 per year for HPV 16,  2.10 per year for HPV 18,  2.10 per year for HPV 31/33/45/52/58 and remaining HR HPV types | [6, 7] |
| $\nu_{16,1}$,$\nu_{18,1}$,$\nu_{nona,1}$, $\nu_{other,1}$ | Progression rate from CIN1 to regressive CIN2/3 | 0.048 per year for HPV 16,  0.00681 per year for HPV 18,  0.0447 per year for HPV 31/33/45/52/58 and remaining HR HPV types | [6, 7] |
| $\nu_{16,2}$,$\nu_{18,2}$,$\nu_{nona,2}$, $\nu_{other,2}$ | Progression rate from CIN1 to non-regressive CIN2/3 | 0.0454 per year for HPV 16,  0.0450 per year for HPV 18,  0.0110 per year for HPV 31/33/45/52/58 and remaining HR HPV types | [6, 7] |
| $\mu_{16}$, $\mu_{18}$,$\mu_{nona}$, $\mu_{other}$ | Rate of waning natural immunity | 0.0407 per year for HPV 16,  0.0287 per year for HPV 18,  0.0320 per year for HPV 31/33/45/52/58 and remaining HR HPV types | [6, 7] |
| ${VE}_{16}$, ${VE}_{18}$,${VE}_{cross}$, ${VE}_{other}$ | Vaccine efficacy | **Section A.4** | [13, 14] |

# A.2. Model calibration

The model was calibrated to each of the three countries independently. Model calibration was done in two steps: 1) calibration to sexual behaviour data, detailed in **Section A.2.1**, and 2) calibration to HPV prevalence data, detailed in the **Section A.2.2**.

## A.2.1. Calibration to sexual behaviour data

In the first step, sexual behaviour parameters of the model are derived from sexual behaviour data. The data from the Demographic and Health Survey (DHS) programme in India and Rwanda were used.[10, 11] DHS data for Brazil were relatively old, instead, we used data from a recent survey with coverage over all Brazilian regions.[12]

The age-specific mixing matrix of women $\rho^{W,age}(a,a')$ for each index age $a$ (each row) was parametrized through a beta distribution, following the implementation of reference [15], with mean and standard deviation of age difference estimated from the surveys’ data on age difference between sexual partners by age in women. See **Figure A3** for the derived mixing patterns. The mixing matrix in men $\rho^{M,age}\left( a,a^{'} \right)$ was obtained by transposing the mixing matrix of women and subsequently reweighting each row to unity.

| **A. India** **** |
| --- |
| **B. Rwanda**   |
| **C. Brazil**  **** |

**Figure A3. Mixing patterns in women at selected ages derived from sexual behaviour survey data**.

Parametrization of sex-, age-, and CSA-specific partner acquisition rates $c^{g}(a, l)$ was done as follows. First, we derived the sex- and age-specific partner acquisition rate $c^{g}(a)$ based on the reported number of sexual partners in each of the surveys. See **Figure A4** for the derived smoothed partner acquisition rates. Then, we made these rates also CSA-specific by applying rate ratios between different CSAs. To derive rate ratios between different CSA we used the non-age-specific the number of sex partners including spouse in the last 12 months. We defined the distribution of population in the three CSAs, which was set to be $\{p^{g}\left( low \right),p^{g}\left( medium \right),p^{g}\left( high \right)\}=\{0.80, 0.15, 0.05\}$ and back-calculated the CSA-specific rates that would match the observed distribution of the data using a Markov Chain Monte Carlo procedure.[15] The ratios between these CSA-specific rates were then used to obtain $c^{g}(a,l)$.

See **Table A1** for the values and references of all fixed and data-derived parameters related to sexual behaviour.

| 1. **India**   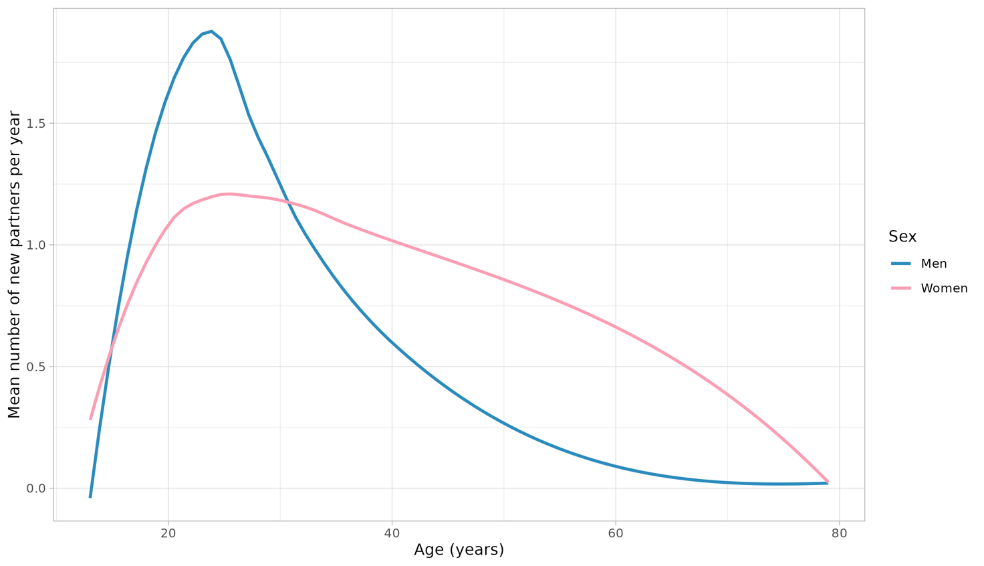 |
| --- |
| 1. **Rwanda**   **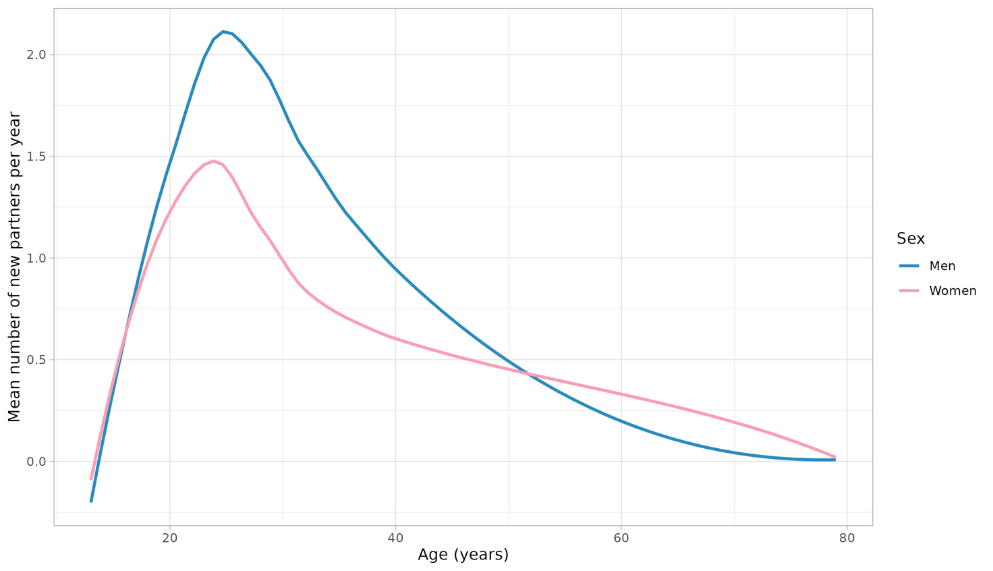** |
| 1. **Brazil**   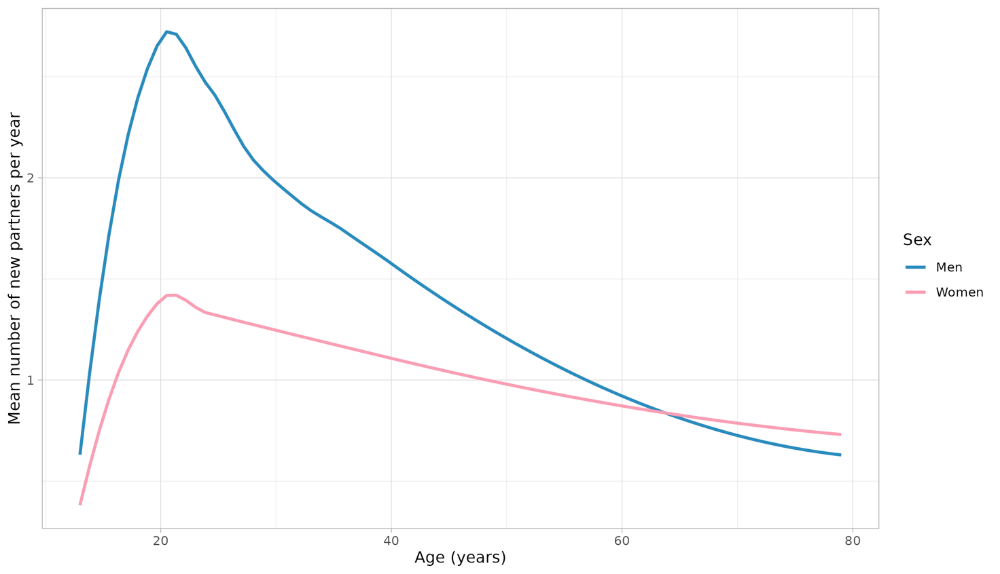 |

**Figure A4. Partner acquisition rates derived from sexual behaviour survey data**.

## A.2.2. Calibration to HPV prevalence data

In the second step of the calibration procedure, remaining sexual behaviour parameters and HPV natural history parameters of the model were calibrated to fit the HPV prevalence data. This was done by fitting the HPV prevalence among women in the model to observed HPV prevalence data.[12, 16-18]

Due to the low prevalence of some HR HPV types, we used the average prevalence of the following four groups of HR HPV types as target prevalence: HPV 16 ($i=16$), HPV 18 ($i=18$), HPV 31/33/45/52/58 ($i=nona$, i.e., HR types covered by the nonavalent vaccine), HPV 35/39/51/56/59/68 ($i=other$). We chose to group HPV 31/33/45 ($i=cross$) and HPV 52/58 together to ensure stable prevalence estimates. In the subsequent step of simulating the impact of a quadrivalent vaccine, we then simulate different copies of the model to model cross-protection for HPV 31/33/45 and no cross-protection for HPV 52/58.

The parameter values regarding the type-specific progression, clearance rates and waning rates of natural immunity were fixed to those estimated for the extensively validated cervical cancer progression model.[6, 7] The parameter values obtained in this calibration step consist of four type-specific transmission probabilities $\beta_{i}$ and four assortativeness adjustment parameters $\varepsilon^{W, age}$, $\varepsilon^{M, age},$ $\varepsilon^{W, csa},$and $\varepsilon^{M, csa}$. See **Table A1** for the values and references of all fixed parameters and ranges for the calibrated parameters. Hundred best-fitting parameter sets were obtained through an Approximate Bayesian Computation scheme with the Lenormand method using R package *EasyABC*.[19] Uniform prior distributions were chosen with range reported in **Table A1**. Log-likelihood of the observed HPV prevalence data given the simulated HPV prevalence under a binomial distribution was used as target statistics with an acceptance threshold of 0.05. For each parameter set, log-likelihood was computed at year 150 of simulation after the burn-in period after the model prevalence has stabilized. See **Figure A5** for the fit to the HPV target prevalence and **Figure A6** for the posterior distribution of the calibrated parameters.

Overall, the model fit was satisfactory. **Figure A5** shows that the model prevalence of HPV 16 in the youngest age group of age 15-19 for India and Rwanda is slightly lower than the observed prevalence (although still within the confidence intervals). Due to the low sample size in this age group (hence the wide confidence intervals) and the selection in the HPV prevalence surveys for sexually active women, we regard the prevalence at older age groups more important for the overall model fit. The infections at older age, which are the ones that persist, are those that will eventually turned into cervical cancer. In contrast, most infections in the youngest age group are transient infections and will be clearly within a few years. Nevertheless, if the model actually underestimates the prevalence of HPV 16 in young age, it might have led to an overestimation of the impact of catch-up vaccination.

| **A. India** **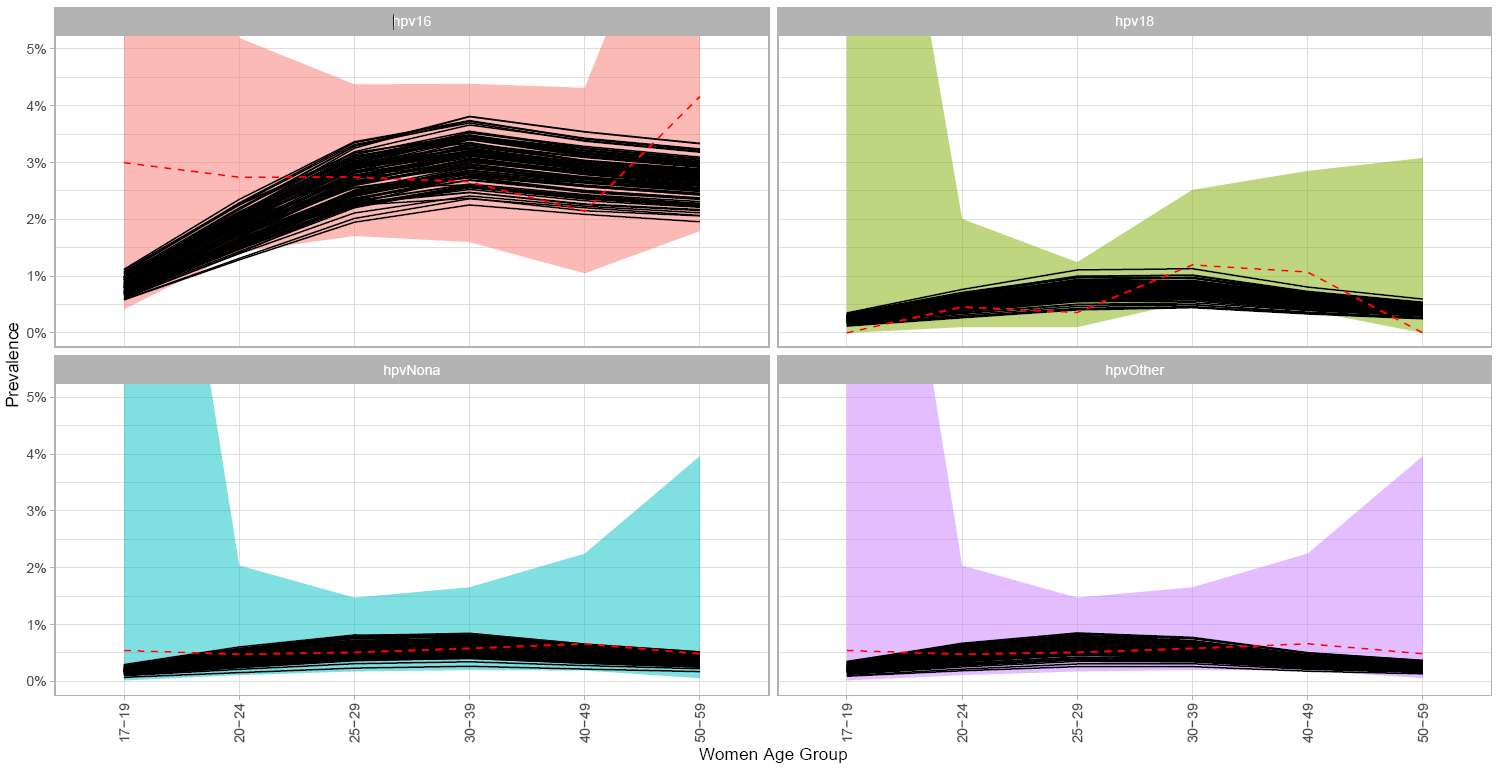** |
| --- |
| **B. Rwanda**  **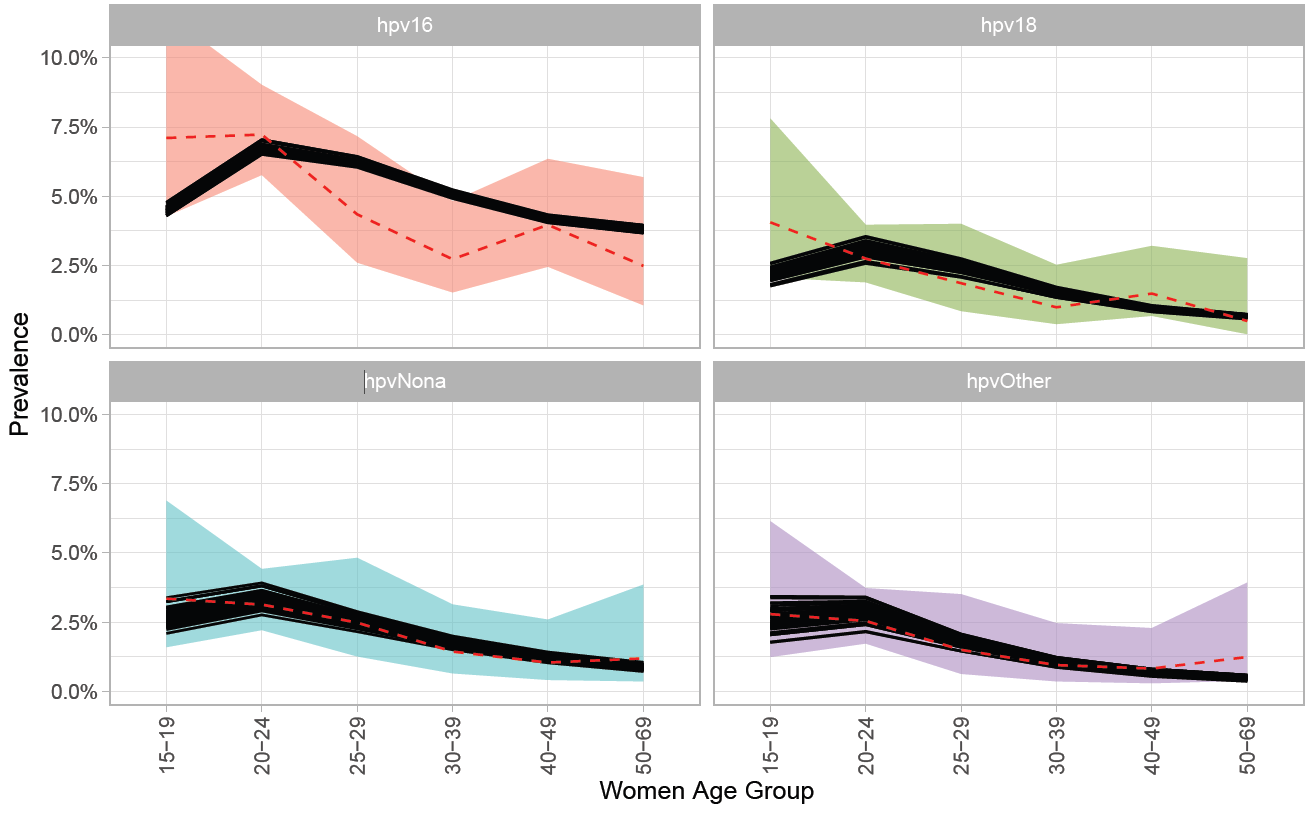** |
| **C. Brazil**  **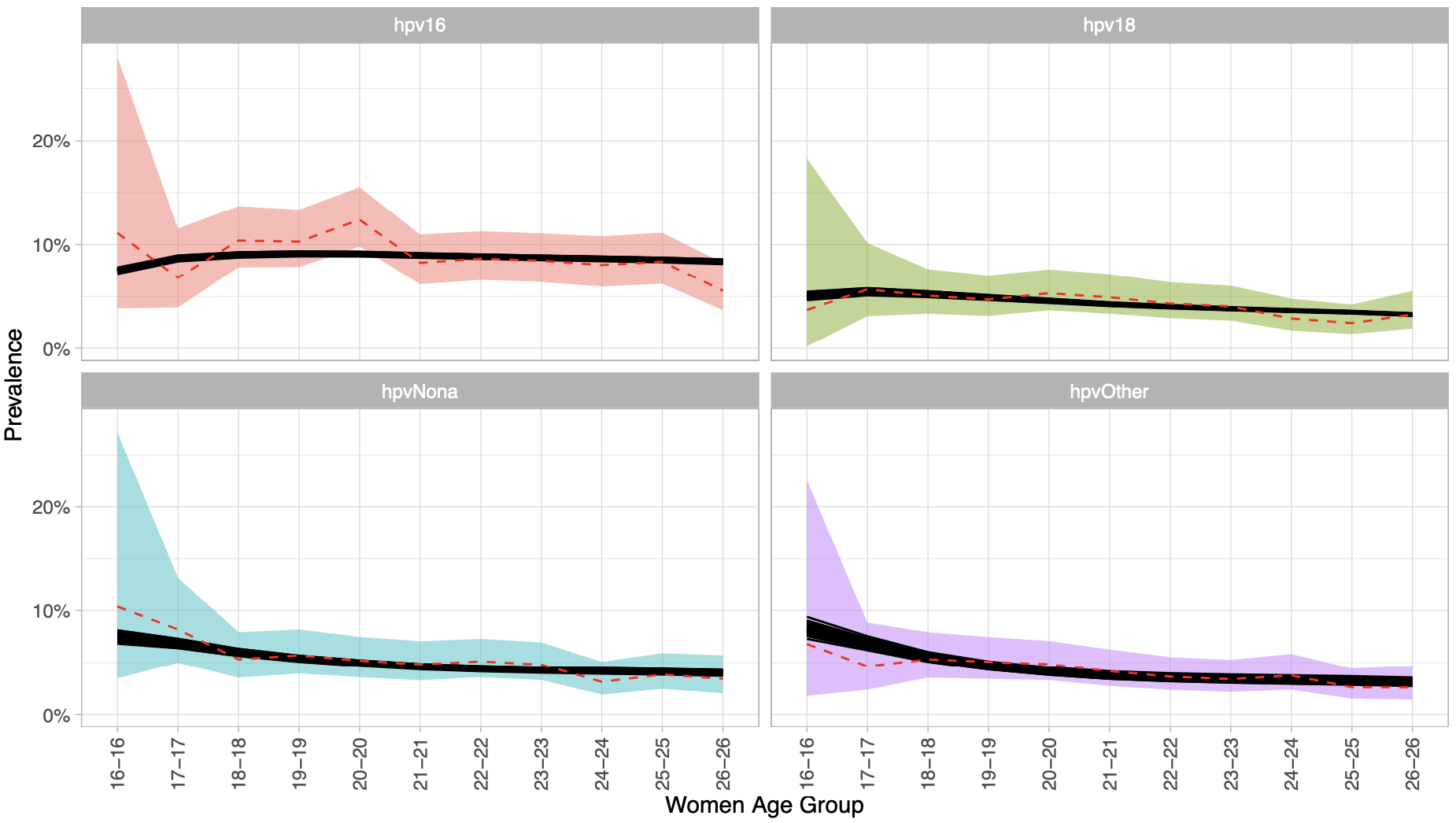** |

**Figure A5. Model fit of the type-specific HPV prevalence data**. Model estimates of HPV prevalence of each of the 100 best-fitting parameter sets are given by a separate line. The point estimates and the 95% confidence intervals of the observed HPV prevalence under binomial distribution are given by the red dashed line and the coloured shade, respectively.

| **A. India** **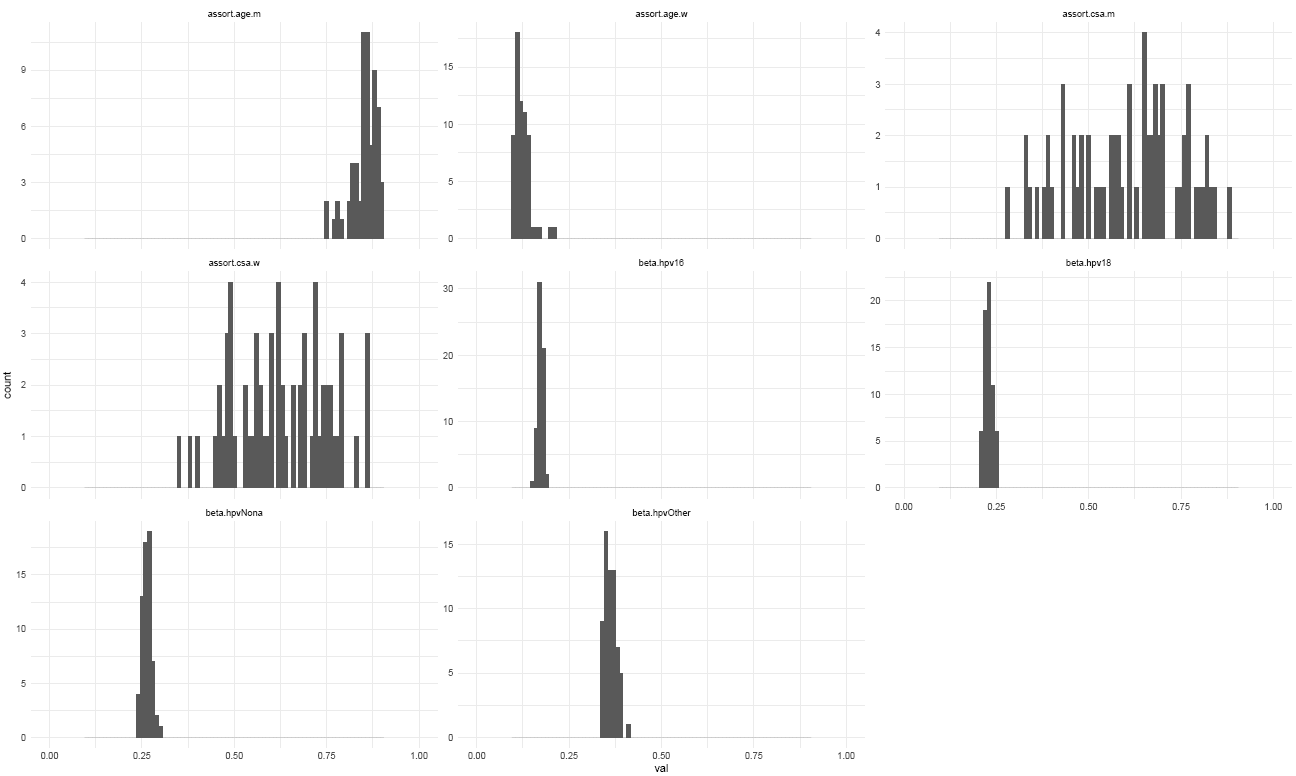** |
| --- |
| **B. Rwanda**  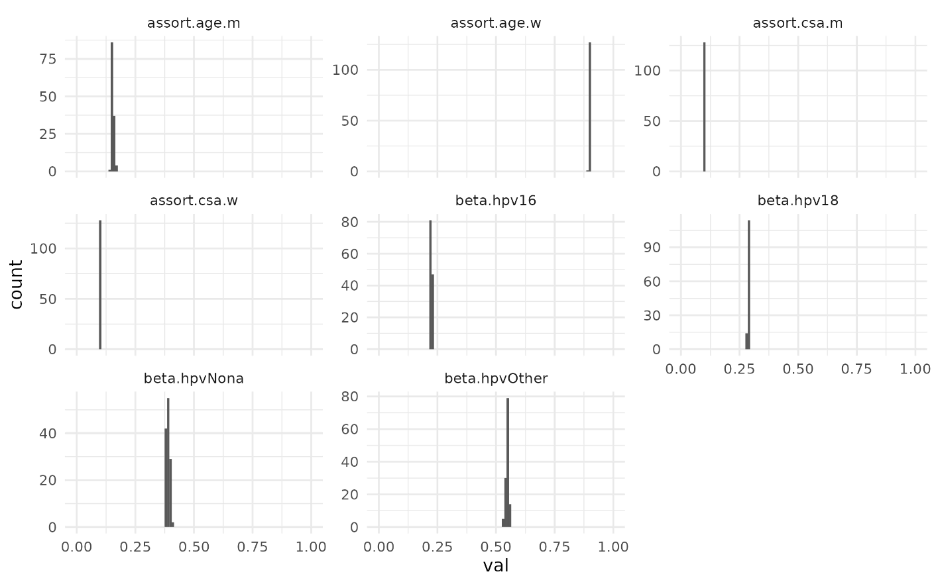 |
| **C. Brazil**  **** |

**Figure A6. Calibrated assortativeness parameters and transmission probabilities.** The value of the age assortativeness adjustment parameters $\varepsilon^{g, age}$ should be between zero and one. When being one, mixing is completely proportionate, and when being zero, mixing occurs according to the data-derived mixing matrices. As for the CSA assortativeness adjustment parameters $\varepsilon^{g, csa}$, being one is completely proportionate, and being zero is mixing only within each CSA.

# A.3. Computation of model outcomes

## A.3.1. Impact on HPV cumulative incidence

For each of model HPV type $i$ and each birth cohort, model estimates on half-year incidence $r_{i,a}$up to age $a=40$ years were combined to derive the cumulative risk of HPV infection $Y_{i}$, as follows: $Y_{i}=\sum_{a=15}^{45} 0.5r_{i,a}s_{a}$. Here, $s_{a}=\exp(-\sum_{a'=10}^{4} 0.5d_{a'})$ is the survival probability up to age $a$ derived from the mortality rates $d_{a'}$ of the UN mortality rates (**Table A2**).[9] Impact estimates were derived as the average of the 100 best-fitting parameter sets.

## A.3.2. Impact on number of cervical cancer cases

A previously published cervical cancer progression model, Atlas, [20] was to derive the number of new cervical cancer cases due to cervical cancer from age-specific cervical cancer incidence data from GLOBOCAN 2022 (**Table A3**).[21] This was first done for the counter-factual scenario without vaccination. This method converts age-specific cancer incidence into cases of cervical cancer (in cases per 100,000 women born) while accounting for the competing risk of dying from other causes before possible occurrence of cervical cancer. UN mortality data were used for the female competing rate of other causes of death (**Table A2**).[9] Outcomes were stratified by age and cohort.

To derive the number of cervical cancer cases by age in scenarios with vaccination, we approximated the relative reduction in risk of cervical cancer by RHEA-estimates of the relative reduction in the cumulative incidence, weighted by HPV-type-specific attributable fraction in cervical cancer, estimated in a recent systematic review by IARC (**Table A4**).[22] Finally, we scaled the obtained number of cases per 100,000 women born to the population size as given by UN data.

**Table A2. Female all-cause mortality rates.**Annual mortality rates by 5-year age group based on UN 2021 estimates.[23]

| **Age group** | **India** | **Rwanda** | **Brazil** |
| --- | --- | --- | --- |
| 0-0 | 0.03725 | 0.02721 | 0.02038 |
| 1-4 | 0.00377 | 0.00226 | 0.00105 |
| 5-9 | 0.00172 | 0.00070 | 0.00040 |
| 10-14 | 0.00105 | 0.00056 | 0.00032 |
| 15-19 | 0.00149 | 0.00090 | 0.00052 |
| 20-24 | 0.00188 | 0.00125 | 0.00074 |
| 25-29 | 0.00221 | 0.00164 | 0.00091 |
| 30-34 | 0.00259 | 0.00219 | 0.00113 |
| 35-39 | 0.00329 | 0.00309 | 0.00151 |
| 40-44 | 0.00438 | 0.00439 | 0.00217 |
| 45-49 | 0.00588 | 0.00639 | 0.00337 |
| 50-54 | 0.00891 | 0.00950 | 0.00514 |
| 55-59 | 0.01289 | 0.01389 | 0.00794 |
| 60-64 | 0.02027 | 0.02177 | 0.01277 |
| 65-69 | 0.03243 | 0.03409 | 0.02195 |
| 70-74 | 0.05162 | 0.05453 | 0.03833 |
| 75-79 | 0.07995 | 0.08460 | 0.06649 |
| 80-84 | 0.12665 | 0.13466 | 0.11541 |
| 85-89 | 0.19814 | 0.21291 | 0.18635 |
| 90-94 | 0.27411 | 0.30334 | 0.28604 |
| 95-99 | 0.37180 | 0.41690 | 0.40918 |

**Table A3. Cervical cancer incidence.**

Incidence in cases per 100,000 women-years by 5-year age groups based on GLOBOCAN 2022 estimates.[21]

| **Age group** | **India** | **Rwanda** | **Brazil** |
| --- | --- | --- | --- |
| 10-14 | 0.02 | 0.00 | 0.00 |
| 15-19 | 0.03 | 0.14 | 0.16 |
| 20-24 | 0.00 | 0.00 | 1.50 |
| 25-29 | 2.46 | 1.83 | 7.03 |
| 30-34 | 8.71 | 7.58 | 12.12 |
| 35-39 | 16.71 | 16.42 | 16.85 |
| 40-44 | 27.48 | 30.77 | 21.08 |
| 45-49 | 39.06 | 46.53 | 25.12 |
| 50-54 | 50.55 | 61.54 | 29.33 |
| 55-59 | 58.71 | 70.28 | 33.87 |
| 60-64 | 64.25 | 73.45 | 38.23 |
| 65-69 | 65.84 | 66.46 | 42.16 |
| 70-74 | 65.61 | 55.48 | 45.35 |
| 75-79 | 64.75 | 45.25 | 47.37 |
| 80-84 | 62.73 | 34.40 | 48.50 |

**Table A4. HPV-type-specific attributable fractions to cervical cancer.**Attributable fraction based on Wei et al.[22]

| **HPV type** | **India** | **Rwanda** | **Brazil** |
| --- | --- | --- | --- |
| 16 | 0.714 | 0.571 | 0.639 |
| 18 | 0.135 | 0.166 | 0.157 |
| 31/33/45 | 0.089 | 0.186 | 0.119 |
| 52/58 | 0.023 | 0.032 | 0.037 |
| Other types | 0.039 | 0.045 | 0.048 |

# A.4. Single-dose vaccine protection scenarios

In the model, vaccine-induced protection is assumed to impede acquisition of new HPV infections. The vaccine-induced protection is assumed to follow an all-or-nothing mechanism, meaning that a vaccinated individual is either fully protected or not protected at all. Vaccine efficacy is here defined as the proportion of the vaccinated individuals that are fully protected. Waning of vaccine protection is modelled as a decrease in vaccine efficacy, meaning a decrease of the proportion of individuals that are fully protected turning into not protected at all. Vaccine efficacy was assumed to decrease according to an exponential decay with possible plateau above 0%, i.e., following the following parametric form: $\left( {VE}_{initial}-{VE}_{plateau} \right)*e^{-rate*time}+{VE}_{plateau}$, with $time$ in years.

Initially, we derived five scenarios of vaccine protection for single-dose vaccination A-E (**Table A5**) in previous publication using efficacy and immunogenicity data from the IARC India vaccine trial.[13, 14] See Appendix A.4.2 of our earlier publication for the exact description of the derived scenarios.[24] However, newer data from the KEN SHE trial indicate a lower bound as high as 90% for single-dose vaccine efficacy in the first three years, making obsolete the two most pessimistic scenarios D and E. Hence, we only considered scenarios A-C in this study.[25]

**Table A5. Overview of parameters under different vaccine protection scenarios.**

| **Scenarios** | **HPV 16** | | | **HPV 18** | | | **HPV 31/33/45** | | |
| --- | --- | --- | --- | --- | --- | --- | --- | --- | --- |
|  | ${VE}_{initial}$ | ${VE}_{plateau}$ | $rate$ | ${VE}_{initial}$ | ${VE}_{plateau}$ | $rate$ | ${VE}_{initial}$ | ${VE}_{plateau}$ | $rate$ |
| A | 0.95 | 0.95 | NA | 0.95 | 0.95 | NA | 0.09 | 0.09 | NA |
| B | 0.95 | 0.60 | 0.02 | 0.95 | 0.45 | 0.02 | 0.09 | 0.45/0.95*0.09 | 0.02 |
| C | 0.90 | 0.55 | 0.02 | 0.85 | 0.35 | 0.04 | 0.09 | 0.35/0.85*0.09 | 0.04 |
| ~~D~~ | ~~0.85~~ | ~~0.50~~ | ~~0.02~~ | ~~0.55~~ | ~~0.25~~ | ~~0.08~~ | ~~0.09~~ | ~~0.25/0.55*0.09~~ | ~~0.08~~ |
| ~~E~~ | ~~0.85~~ | ~~0.50~~ | ~~0.02~~ | ~~0.55~~ | ~~0.25~~ | ~~0.08~~ | ~~0~~ | ~~0~~ | ~~NA~~ |

| 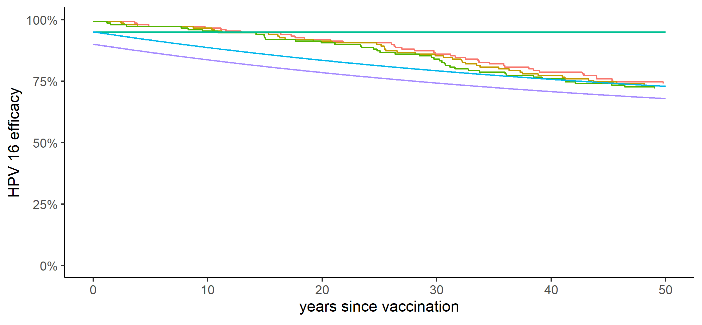**A** | 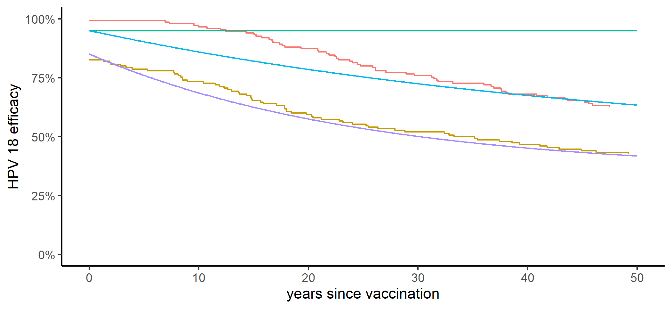**B** |
| --- | --- |
| 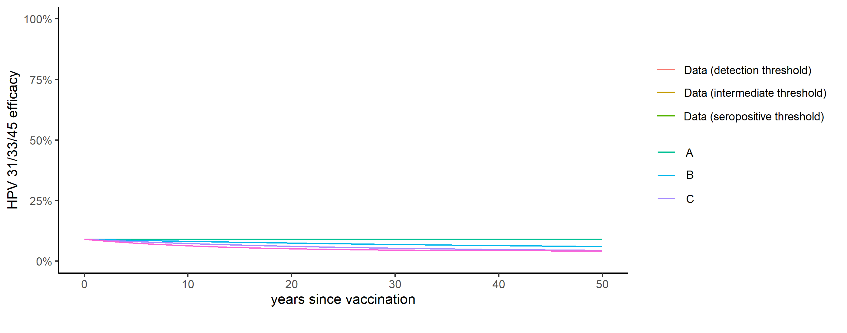**C** | |

**Figure A7. Scenarios of single-dose vaccine efficacy by HPV type.**

# A.5. HPV-FRAME checklist

**Table A6. HPV-FRAME checklist. [26]**

| **Core reporting standard** |  |  |  |
| --- | --- | --- | --- |
| **a) Inputs** | **Reported by age?**  **(Y/N)** | **Report by sex?**  **(F-only, M-only or both)** | **Comments** |
| Target population for intervention | Yes | Both | Vaccination in girls and boys in routine programme and catch-up in older female cohorts were considered. |
| Sexual behaviour | Yes | Both | Derivation of the following parameters from surveys reported: age- and sex-specific partnership acquisition rates; age- and sex-specific age mixing matrices. Calibration of assortativeness parameters and type-specific probability of HPV transmission were reported. |
| Cohort examined for evaluation / time horizon | No | Female only | Lifetime risk of cervical cancer was presented for the cohorts (some vaccinated, some unvaccinated) aged 11-30 at 2025. Long-term age-standardized incidence rate of cervical cancer was reported at 100 years after the introduction of vaccination. |
| Quality of life assumptions | NA | NA | NA |
| Calibration | Yes | Both | Approximate Bayesian Computation scheme with the Lenormand method using R package EasyABC was used to obtain age-, sex, and risk-groups assortativeness parameters and type-specific probability of HPV transmission. Log-likelihood of the observed HPV prevalence data given the simulated HPV prevalence under a binomial distribution was used as target statistics. |
| Validation (where possible) | NA | NA | NA |
| Costs | NA | NA | NA |

(Table continues the next page)

| **Reporting standard for HPV vaccination in adolescent individuals** | | | | |
| --- | --- | --- | --- | --- |
| **a) Inputs** | **Reported?  (Y/N)** | **Reported by age? (Y/N)** | **Report by sex?  (F-only, M-only or both)** | **Report as calibration or validation target? (Y/N)** |
| Vaccine uptake | Yes | Yes | Both | The historical sex- and cohort-specific uptake was reconstructed and reported. Simulated scenarios of possible sex- and cohort-specific uptake after 2025 for both catch-up and routine vaccination were reported. |
| Vaccine efficacy | Yes | NA | NA | Efficacy by dose schedule and HPV type were considered. Efficacy was independent of age and sex. |
| Vaccine cross-protection | Yes | NA | NA | Level of cross-protection for HPV 31/33/45 was reported. Efficacy was independent of age and sex. |
| Duration vaccine protection and waning | Yes | Yes | NA | Waning assumption by dose schedule and HPV type was considered. |
| Vaccine and delivery costs | Yes | No | NA | A crude estimated of resource saving in terms of vaccine and delivery costs was reported. Data on delivery costs by age were not available to be incorporated. No comprehensive economic assessment was done in this paper. |
| Pre-vaccination disease burden (including population attributable fractions for HPV) | Yes,  for cervical cancer. | Yes,  for cervical cancer. | Female only,  for cervical cancer. | Country-specific attributable fraction by HPV type to cervical cancer burden was reported. |
| Duration of natural immunity | Yes | NA | Both | Natural immunity was independent of age and only present for women. Sex-specific natural immunity assumptions were reported. |
| **b) Outputs** | **Reported?  (Y/N)** | **Reported by age? (Y/N)** | **Report by sex? (F-only, M-only or both)** | **Comments** |
| Absolute reductions in HPV infections, and/or warts, post-vaccination | No | NA | NA | Model-estimates of HPV infection reduction were used to project reduction in cervical cancer risk but not reported. |
| Absolute reductions in CIN2+ post-vaccination | No | NA | NA | NA |
| Absolute reductions in invasive cancer (cervical and other HPV cancers, as relevant) | Yes,  for cervical cancer. | No | Female only,  cervical cancer. | Model-estimates of cervical cancer risk reduction were reported. |

| **Reporting standards for evaluations of vaccination at older ages** | | | | |
| --- | --- | --- | --- | --- |
| **a) Inputs** | **Detail** | **Reported by age? (Y/N)** | **Report by sex? (F-only, M-only or both)** | **Comments** |
| Natural history | Nature history structure used in the model (including progression and regression from high grade to low grade disease/productive HPV infection). | NA | Yes | Natural history structure was dependent on the time since infection acquisition but not on age. Sex-specific natural history parameters were reported. |
| Natural history | Rate of clearance of HPV infection | NA | Yes | Rate of clearance of HPV infection dependent on the time since infection acquisition but not on age. Sex-specific natural history parameters were reported. |
| Natural history | Rate of loss of naturally acquired immunity | NA | Yes | Rate of loss of naturally acquired immunity was independent of age. Naturally acquired immunity was only assumed for women and reported. |
| Natural history | Simulation of latency by HPV type (handling of apparently new infections in older women – is the possibility that some are reactivated latent infections explored in sensitivity analysis?) | NA | NA | No latency assumed in the model. |
| Vaccination | Vaccine coverage at older ages | Yes | NA | Catch-up at older ages was only considered for women and reported. |
| Vaccination | Whether screen-and-vaccinate is being modelled, or just vaccination at older ages (without linking to screening/HPV status) | NA | NA | We did not model screen-and-vaccinate. We modelled vaccination at older ages but without linking to screening/HPV status. |

| **Reporting standard for models of HPV prevention in LMIC** | | |  |  |
| --- | --- | --- | --- | --- |
| **a) Inputs** | **Reported? (Y/N)** | **Reported by age? (Y/N)** | **Report by sex?  (F-only, M-only or both)** | **Comments** |
| HIV prevalence rates if endemic in country | Yes | No | No | HIV prevalence in the three modelled countries was reported in the appendix. The effects of HIV are not modelled because of the low HIV prevalence. |
| Description of any opportunistic or pilot/demonstration screening project ongoing | Yes | NA | NA | Brief description of the countries’ screening programme was reported in Table 1. |

| **Reporting standards for evaluations assessing alternative vaccine types or reduced-dose schedules** | | | | |
| --- | --- | --- | --- | --- |
| **a) Inputs** | **Reported? (Y/N)** | **Reported by age? (Y/N)** | **Report by sex?  (F-only, M-only or both)** | **Comments** |
| Vaccine efficacy/waning | See Comments. | See Comments. | See Comments. | See entries "Vaccine efficacy" and "Duration vaccine protection and waning" under "Reporting standard for HPV vaccination in adolescent individuals". |
| Timing between doses (for 2-dose) | NA | NA | NA | Timing between the two doses under two-dose vaccination schedule was not modelled. We assumed constant and lifelong efficacy under two-dose schedule. |
| Vaccine cross-protection | See Comments. | See Comments. | See Comments. | See entries "Vaccine cross-protection" under "Reporting standard for HPV vaccination in adolescent individuals". |
| Cost | See Comments. | See Comments. | See Comments. | See entries "Vaccine and delivery costs" under "Reporting standard for HPV vaccination in adolescent individuals". |
| **b) Outputs** | **Reported? (Y/N)** | **Reported by age? (Y/N)** | **Report by sex? (F-only, M-only or both)** | **Report as calibration or validation target (Y/N)?** |
| Threshold cost per dose | NA | NA | NA | No cost-effectiveness analyses performed. |

**Appendix B**

# B.1. Supplementary results in figures

**Figure B1. Impact of additional female catch-up in 2025 on lifetime cervical cancer risk in waning scenarios.**

| **A. Single-dose protection scenario A (same as Figure 1)**  **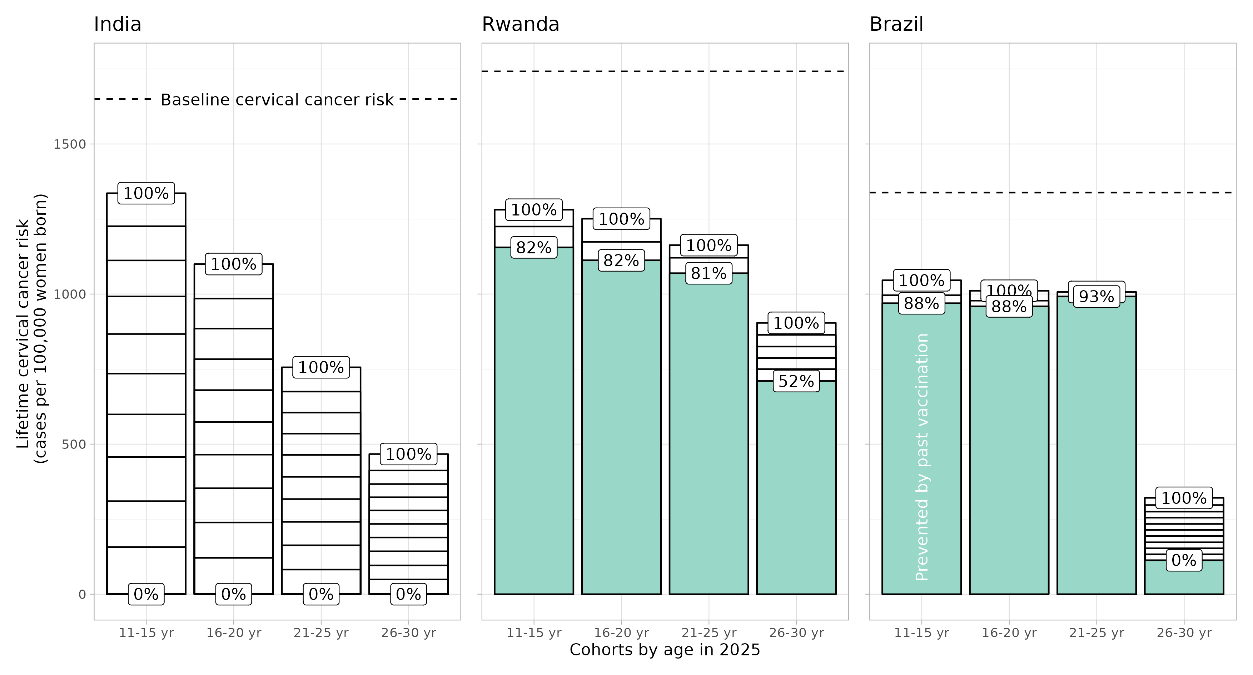** |
| --- |
| **B. Single-dose protection scenario B**  **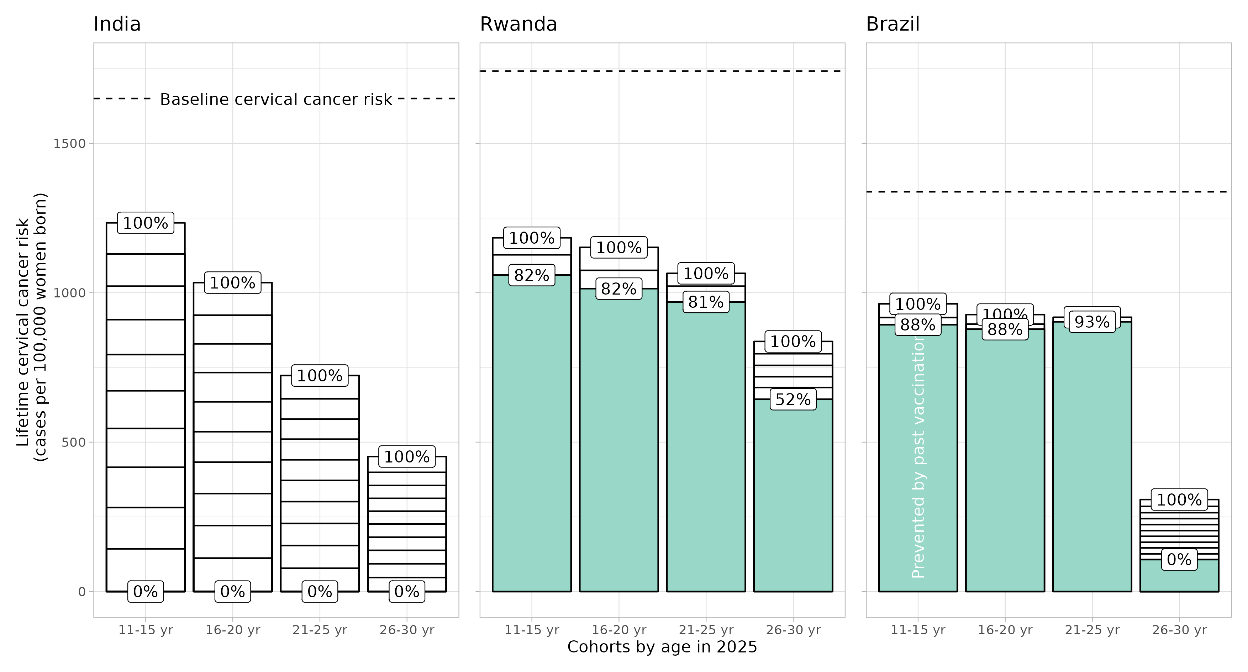** |
| **C. Single-dose protection scenario C**  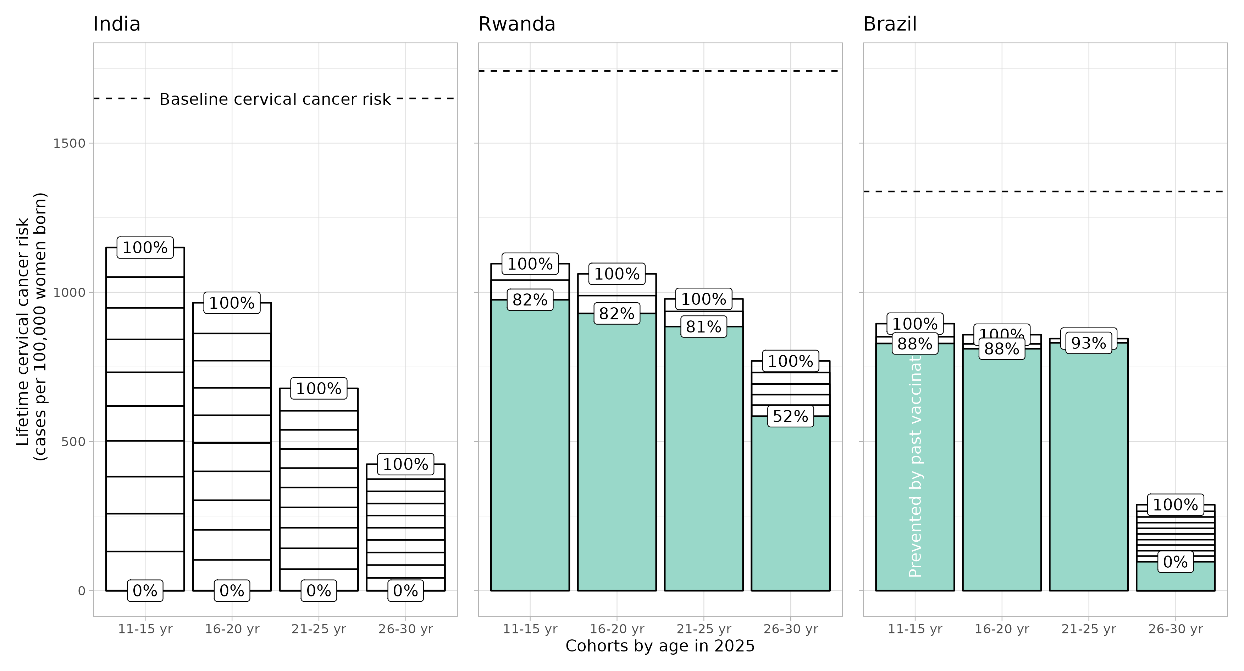 |

**Figure B2. Expected long-term ASIR among for combinations of coverage in girls and boys in waning scenarios.**


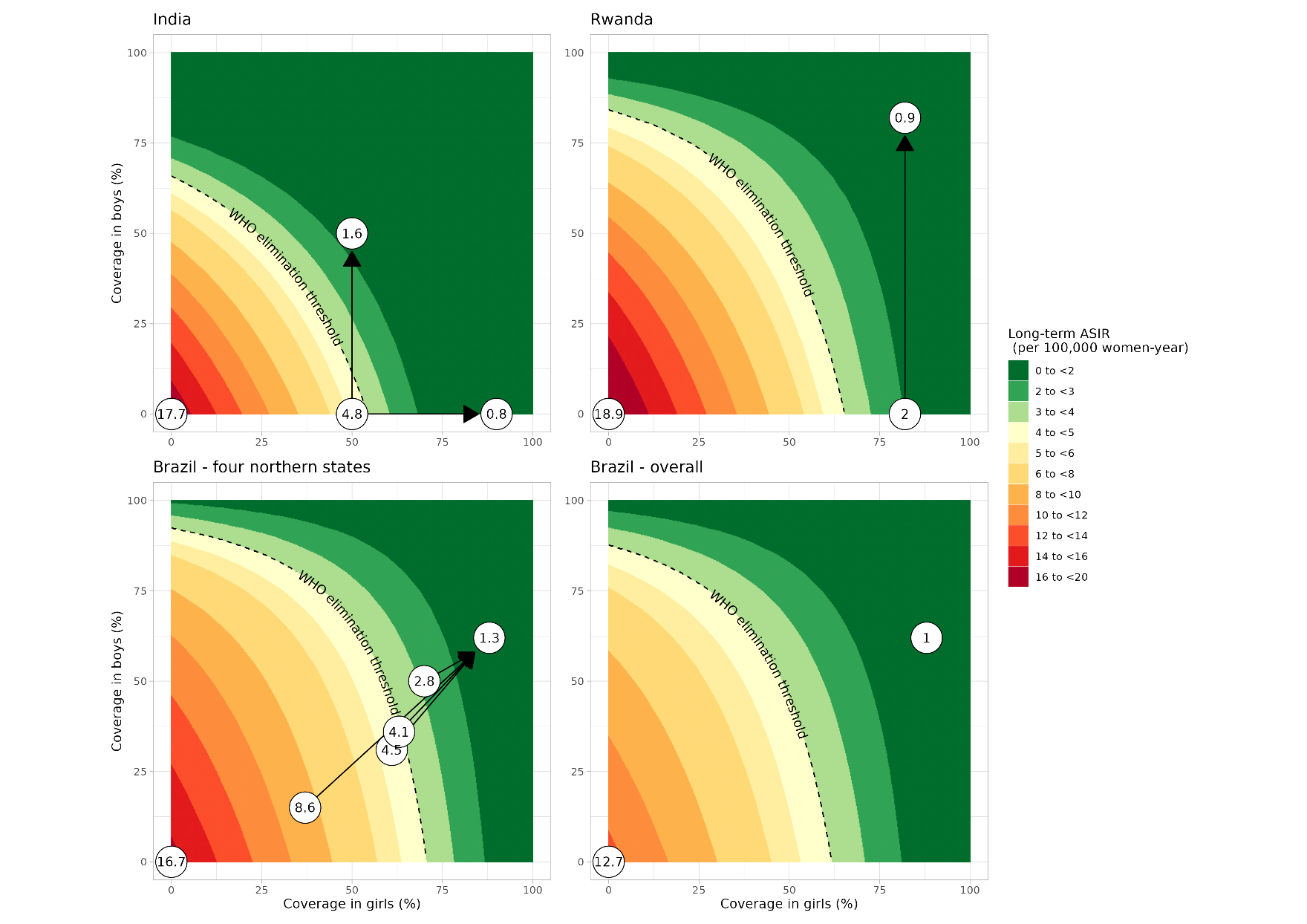


**Figure B3. Expected long-term ASIR among for combinations of coverage in girls and boys in waning scenarios.**

| **A. Single-dose protection scenario A  (same as Figure 2)**  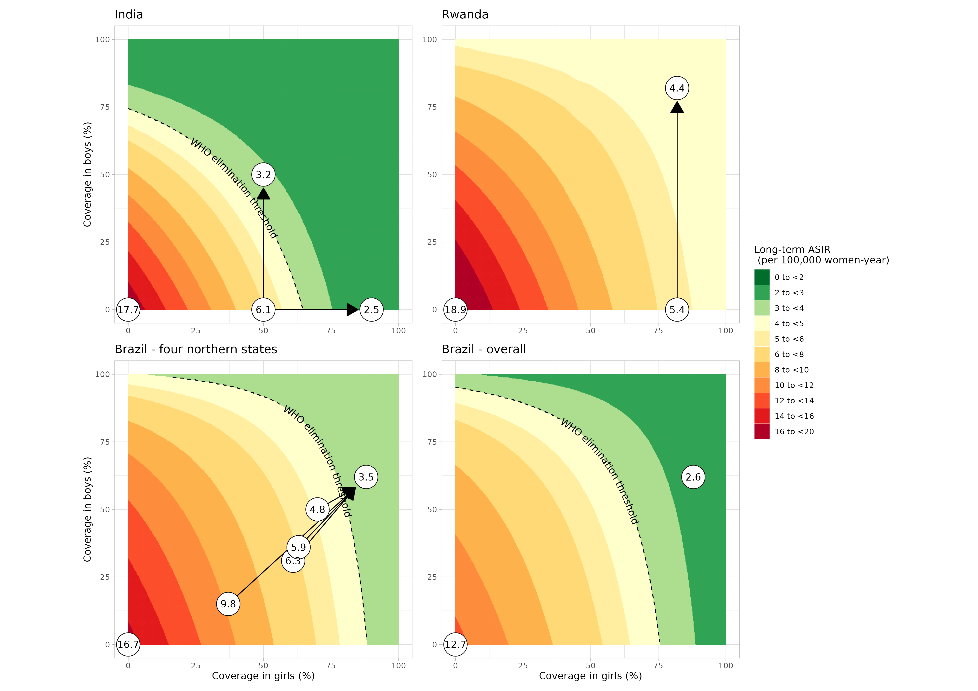 | **B. Single-dose protection scenario B**  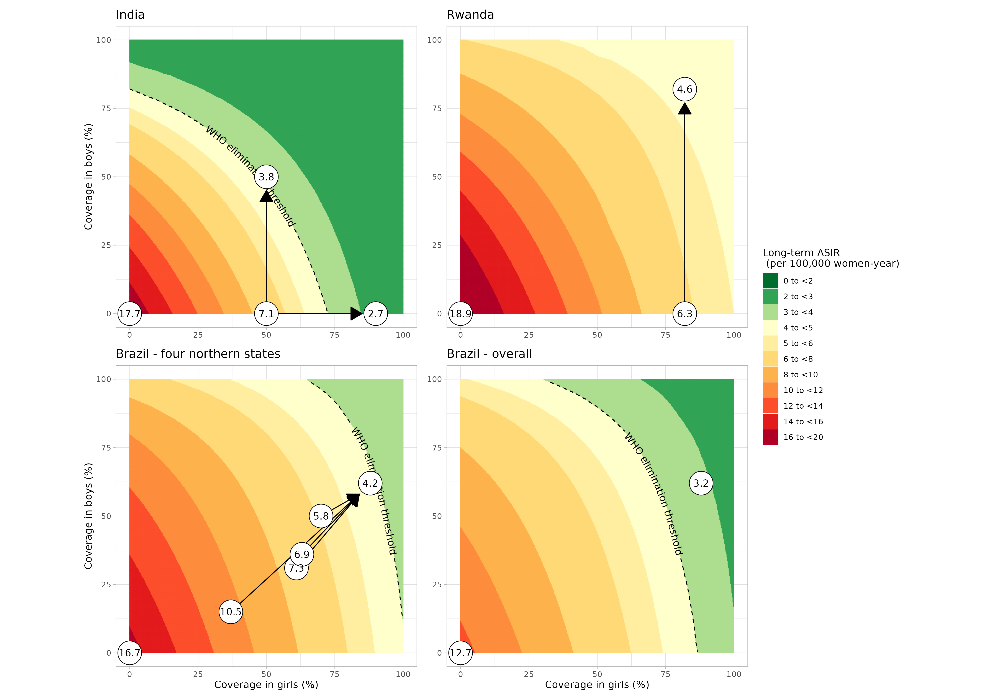 | **C. Single-dose protection scenario C**  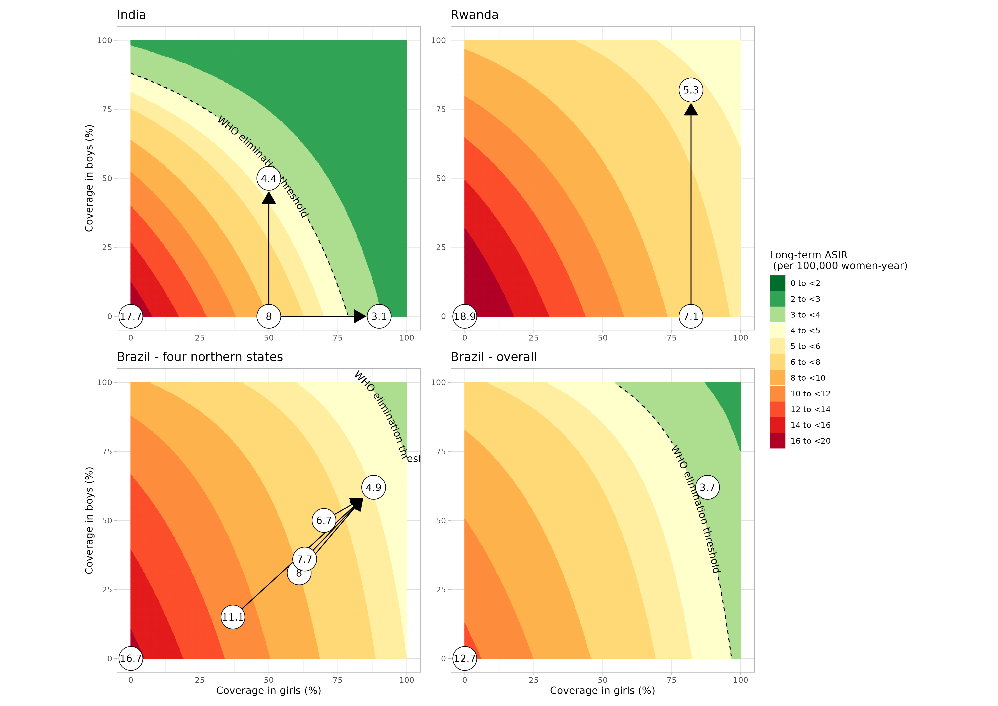 |
| --- | --- | --- |

# Reference

1. Baussano I, Elfström KM, Lazzarato F, Gillio-Tos A, De Marco L, Carozzi F, et al. Type-specific human papillomavirus biological features: Validated model-based estimates. PLoS ONE. 2013;8(11):e81171. doi: 10.1371/journal.pone.0081171.

2. METHIS platform by Public Health Decision Modeling Team [cited 2023 July 25]. Available from: <https://iarc-miarc.gitlab.io/methis/methis.website/>.

3. Giuliano AR, Viscidi R, Torres BN, Ingles DJ, Sudenga SL, Villa LL, et al. Seroconversion Following Anal and Genital HPV Infection in Men: The HIM Study. Papillomavirus Res. 2015;1:109-15. Epub 2015/12/22. doi: 10.1016/j.pvr.2015.06.007. PubMed PMID: 26688833; PubMed Central PMCID: PMCPMC4680989.

4. Schiffman M, Doorbar J, Wentzensen N, de Sanjosé S, Fakhry C, Monk BJ, et al. Carcinogenic human papillomavirus infection. Nat Rev Dis Primers. 2016;2:16086. Epub 2016/12/03. doi: 10.1038/nrdp.2016.86. PubMed PMID: 27905473.

5. Giuliano AR, Lazcano-Ponce E, Villa LL, Flores R, Salmeron J, Lee JH, et al. The human papillomavirus infection in men study: human papillomavirus prevalence and type distribution among men residing in Brazil, Mexico, and the United States. Cancer Epidemiol Biomarkers Prev. 2008;17(8):2036-43. Epub 2008/08/19. doi: 10.1158/1055-9965.Epi-08-0151. PubMed PMID: 18708396; PubMed Central PMCID: PMCPMC3471778.

6. Berkhof J, Bogaards JA, Demirel E, Diaz M, Sharma M, Kim JJ. Cost-Effectiveness of Cervical Cancer Prevention in Central and Eastern Europe and Central Asia. Vaccine. 2013;31(S7):H71-H9. doi: 10.1016/J.VACCINE.2013.04.086.

7. Bogaards JA, Xiridou M, Coupé VMH, Meijer CJLM, Wallinga J, Berkhof J. Model-based estimation of viral transmissibility and infection-induced resistance from the age-dependent prevalence of infection for 14 high-risk types of human papillomavirus. American Journal of Epidemiology. 2010;171(7):817-25. doi: 10.1093/aje/kwp466.

8. Stelzle D, Tanaka LF, Lee KK, Khalil AI, Baussano I, Shah AS, et al. Estimates of the global burden of cervical cancer associated with HIV. The Lancet Global Health. 2020;9(2):e161-e9.

9. United Nations Population Division [cited 2023 15 December]. Available from: <https://population.un.org/dataportal/home?df=001b507f-a061-4265-becf-dd45443b7c91>.

10. International Institute for Population Sciences (IIPS) and ICF. 2021. National Family Health Survey (NFHS-5), 2019-21: India. Mumbai: IIPS. [cited 2023 15 December]. Available from: <https://dhsprogram.com/publications/publication-FR375-DHS-Final-Reports.cfm>.

11. National Institute of Statistics of Rwanda (NISR) [Rwanda], Ministry of Health (MOH) [Rwanda], and ICF. 2021. Rwanda Demographic and Health Survey 2019-20 Final Report. Kigali, Rwanda, and Rockville, Maryland, USA: NISR and ICF. [cited 2023 15 December]. Available from: <https://dhsprogram.com/publications/publication-FR370-DHS-Final-Reports.cfm>.

12. Wendland EM, Villa LL, Unger ER, Domingues CM, Benzaken AS. Prevalence of HPV infection among sexually active adolescents and young adults in Brazil: The POP-Brazil Study. Sci Rep. 2020;10(1):4920. Epub 2020/03/20. doi: 10.1038/s41598-020-61582-2. PubMed PMID: 32188884; PubMed Central PMCID: PMCPMC7080737 consultant for BD, Roche and Qiagen for HPV tests. All other authors have completed and submitted the COI-corresponding author form for Disclosure of Potential Conflicts of Interest, and none were reported.

13. Basu P, Malvi SG, Joshi S, Bhatla N, Muwonge R, Lucas E, et al. Vaccine efficacy against persistent human papillomavirus (HPV) 16/18 infection at 10 years after one, two, and three doses of quadrivalent HPV vaccine in girls in India: a multicentre, prospective, cohort study. The Lancet Oncology. 2021;22(11):1518-29. doi: 10.1016/S1470-2045(21)00453-8.

14. Joshi S, Anantharaman D, Muwonge R, Bhatla N, Panicker G, Butt J, et al. Evaluation of immune response to single dose of quadrivalent HPV vaccine at 10-year post-vaccination. Vaccine. 2023;41(1):236-45. Epub 2022/11/30. doi: 10.1016/j.vaccine.2022.11.044. PubMed PMID: 36446654; PubMed Central PMCID: PMCPMC9792650.

15. Vänskä S, Auranen K, Leino T, Salo H, Nieminen P, Kilpi T, et al. Impact of vaccination on 14 high-risk HPV type infections: a mathematical modelling approach. PLoS One. 2013;8(8):e72088. Epub 2013/09/07. doi: 10.1371/journal.pone.0072088. PubMed PMID: 24009669; PubMed Central PMCID: PMCPMC3756967 following conflicts: ML has received grants for his HPV vaccination studies through his employer University of Tampere, Finland from Merck & Co., Inc. and GSK Biologicals; PN has been working as a consultant for GSK and Sanofi Pasteur-MSD, TK Nationwide effectiveness study of the 10-valtent pneumococcal conjugate vaccine. Collaborative study was mainly funded by GSK; DA, as an employee of Väestöliitto, participated as the principal investigator in research projects of Merck and GSK during the last 5 years. Väestöliitto has been paid for conducting the research. He has given lectures at educational occasions organized by health care and medical companies and participated in various meetings paid by these (Merck, GSK, Bayer, WHO). Membership in the National Expert Group (2008–2011) on HPV Related Disease Prevention: TL HS PN TK DA ML. This does not alter the authors’ adherence to all of the PLOS ONE policies on sharing data and materials.

16. Franceschi S, Rajkumar R, Snijders PJ, Arslan A, Mahé C, Plummer M, et al. Papillomavirus infection in rural women in southern India. Br J Cancer. 2005;92(3):601-6. Epub 2005/01/26. doi: 10.1038/sj.bjc.6602348. PubMed PMID: 15668709; PubMed Central PMCID: PMCPMC2362069.

17. Dutta S, Begum R, Mazumder Indra D, Mandal SS, Mondal R, Biswas J, et al. Prevalence of human papillomavirus in women without cervical cancer: a population-based study in Eastern India. Int J Gynecol Pathol. 2012;31(2):178-83. Epub 2012/02/10. doi: 10.1097/PGP.0b013e3182399391. PubMed PMID: 22317877.

18. Ngabo F, Franceschi S, Baussano I, Umulisa MC, Snijders PJ, Uyterlinde AM, et al. Human papillomavirus infection in Rwanda at the moment of implementation of a national HPV vaccination programme. BMC Infect Dis. 2016;16:225. Epub 2016/05/26. doi: 10.1186/s12879-016-1539-6. PubMed PMID: 27221238; PubMed Central PMCID: PMCPMC4877733.

19. Jabot F, Faure T, Dumoulin N, Albert C. R package EasyABC: Efficient Approximate Bayesian Computation Sampling Schemes [cited 2023 28 July]. Available from: <https://cran.r-project.org/web/packages/EasyABC/index.html>.

20. Bonjour M, Charvat H, Franco EL, Piñeros M, Clifford GM, Bray F, et al. Global estimates of expected and preventable cervical cancers among girls born between 2005 and 2014: a birth cohort analysis. The Lancet Public Health. 2021;6(7):e510-21. doi: 10.1016/s2468-2667(21)00046-3.

21. Ferlay J, Ervik M, Lam F, Laversanne M, Colombet M, Mery L, et al. Global Cancer Observatory: Cancer Today (version 1.1). Lyon, France: International Agency for Research on Cancer 2024 [cited 2024 15 March]. Available from: <https://gco.iarc.who.int/today>.

22. Wei F, Georges D, Man I, Baussano I, Clifford GM. Causal attribution of human papillomavirus types to invasive cervical cancer worldwide: a systematic analysis of the global literature (Manuscript under review). 2024.

23. UN. World Population Prospects 2022. United Nations. Department of Economic Social Affairs. Population Division 2022 [cited 2022 1 December]. Available from: <https://population.un.org/wpp/Download>.

24. Man I, Georges D, de Carvalho TM, Ray Saraswati L, Bhandari P, Kataria I, et al. Evidence-based impact projections of single-dose human papillomavirus vaccination in India: a modelling study. Lancet Oncol. 2022;23(11):1419-29. Epub 2022/09/30. doi: 10.1016/s1470-2045(22)00543-5. PubMed PMID: 36174583; PubMed Central PMCID: PMCPMC9622421 outside of the submitted work. All other authors declare no competing interests.

25. Barnabas RV, Brown ER, Onono MA, Bukusi EA, Njoroge B, Winer RL, et al. Durability of single-dose HPV vaccination in young Kenyan women: randomized controlled trial 3-year results. Nat Med. 2023;29(12):3224-32. Epub 2023/12/05. PubMed PMID: 38049621; PubMed Central PMCID: PMCPMC10719107 and manuscript writing, outside the submitted work. She serves on a Gilead Sciences Data Monitoring Committee for which she receives an honorarium, outside the submitted work. J.M.B. reports personal fees from Gilead Sciences, Janssen and Merck, outside the submitted work; and is an employee of Gilead Sciences, outside of the submitted work. C.C. reports personal fees from Gilead Sciences and Merck, outside the submitted work. N.M. reports grant support from Merck Pharmaceuticals, outside the submitted work. D.A.G. reports personal fees from Merck, outside the submitted work. E.A.B. reports personal fees from Gilead Sciences, Merck and ViiV, outside the submitted work. R.S.M. reports personal fees from Lupin Pharmaceuticals and donated testing STI testing supplies from Hologic Corporation, outside of the submitted work. All other coauthors (M.A.O., B.N., R.L.W., D.A.G., L.F.P., D.D., I.W., C.B., S.K., K.B.H., D.G.K., D.P., S.M., E.R., S.C. and T.T.S.) have nothing to disclose.

26. Canfell K, Kim JJ, Kulasingam S, Berkhof J, Barnabas R, Bogaards JA, et al. HPV-FRAME: A consensus statement and quality framework for modelled evaluations of HPV-related cancer control. Papillomavirus Research. 2019;8:100184.
